# Assessing ecological and physiological costs of melanism in North American *Papilio glaucus* females: two decades of dark morph frequency declines

**DOI:** 10.1111/1744-7917.12653

**Published:** 2019-01-07

**Authors:** J. Mark Scriber

**Affiliations:** ^1^ Department of Entomology Michigan State University East Lansing Michigan USA; ^2^ McGuire Center for Lepidoptera and Biodiversity Florida Museum of Natural History University of Florida Gainesville Florida USA

**Keywords:** climate warming, ecology of melanism, incomplete mimicry, pleiotrophic costs

## Abstract

Polymorphisms for melanic form of insects may provide various selective advantages. However, melanic alleles may have significant/subtle pleiotrophic “costs.” Several potential pleiotrophic effects of the W (=Y)‐linked melanism gene in *Papilio glaucus* L. (Lepidoptera) showed no costs for melanic versus yellow in adult size, oviposition preferences, fecundity, egg viability, larval survival/growth rates, cold stress tolerance, or postdiapause emergence times. Sexual selection (males choosing yellow rather than mimetic dark females) had been suggested to provide a balanced polymorphism in *P. glaucus*, but spermatophore counts in wild females and direct field tethering studies of size‐matched pairs of virgin females (dark and yellow), show that male preferences are random or frequency‐dependent from Florida to Michigan, providing no yellow counter‐advantages. Recent frequency declines of dark (melanic/mimetic) females in *P. glaucus* populations are shown in several major populations from Florida (27.3°N latitude) to Ohio (38.5° N). Summer temperatures have increased significantly at all these locations during this time (1999–2018), but whether dark morphs may be more vulnerable (in any stage) to such climate warming remains to be determined. Additional potential reasons for the frequency declines in mimetic females are discussed: (i) genetic introgression of Z‐linked melanism suppressor genes from *P. canadensis* (R & J) and the hybrid species, *P. appalachiensis* (Pavulaan & Wright), (ii) differential developmental incompatibilities, or Haldane effects, known to occur in hybrids, (iii) selection against intermediately melanic (“dusty”) females (with the W‐linked melanic gene, b+) which higher temperatures can cause.

## Introduction

Insect melanism has frequently been associated with polymorphic forms variously assumed to be selectively advantageous in certain environmental conditions (better crypsis, mimicry advantages, better thermal regulation, earlier emergence times, or increased mating success, increased disease and parasitoid resistance, desiccation tolerance, and more efficient mate signaling; reviewed by Kingsolver, 1987; Gershenson, 1994; Lederhouse *et al*., 1995; Verhoog *et al*., 1996; Wilson *et al*., 2001; True, 2003; Ma *et al*., 2008; Liu *et al*., 2015). In addition to these ecological advantages, the molecular, biochemical, and genetic basis of melanism has also recently received considerable attention (e.g., in the eastern tiger swallowtail butterfly, *Papilio glaucus* L; Koch *et al*., 2000a; Ffrench‐Constant & Koch, 2003; Cong *et al*., 2015; and in *Drosophila*; Wright, 1987; True *et al*., 1999).

It is not clear how melanic alleles impact other phenotypic traits and resulting ecological fitness in insects. The potential physiological and ecological “costs” for melanism remain largely unknown (Dubovskiy *et al*., 2013), but might be significant in their impacts on size or lower tolerance to desiccation (Safranek & Riddiford, 1975), possible reduction in physiological life span (Ohsaki, 2005), or reduced immunocompetency (Stoehr, 2006; Lindsey & Altizer, 2009). For one species of Lepidoptera (*Helicoverpa armigera* (Hübner); Ma *et al*., 2008) melanism was associated with slower development in all life stages, smaller body size, lower fecundity and a lower reproductive rate. Ethier *et al*. (2015) show that, for nitrogen‐limited folivorous insects, melanism may be physiologically costly (see also Zvereva *et al*., 2002; Talloen *et al*., 2004; Punzalan *et al*., 2008). Melanism also appears to be genetically associated with lower gonad mass and reproductive capacity in sand crickets (Roth & Fairbairn, 2013).

Here, some potential pleiotropic effects of the W (=Y)‐linked gene for dark morph melanism (b+) and its Z (=X)‐linked enabler gene (s–) in the North American eastern tiger swallowtail butterfly, *Papilio (=Pterourus) glaucus* (Scriber *et al*., 1996) are evaluated. Previous studies have explained how the thermal landscape (growing degree‐days of summer) may play a major role in constraining the northern range limits of the bivoltine dark morph *Papilio glaucus* through differential genetic introgression of these and other sex‐linked traits across the hybrid zone with the more northern Canadian swallowtail, *Papilio canadensis* (R & J) especially those impacting voltinism and diapause (Rockey *et al*., 1987a,b; Hagen *et al*., 1991; Scriber *et al*., 2008; Ording *et al*., 2010; Scriber, 2011; Scriber *et al*., 2014; Ryan *et al*., 2018a).

**Figure 1:**
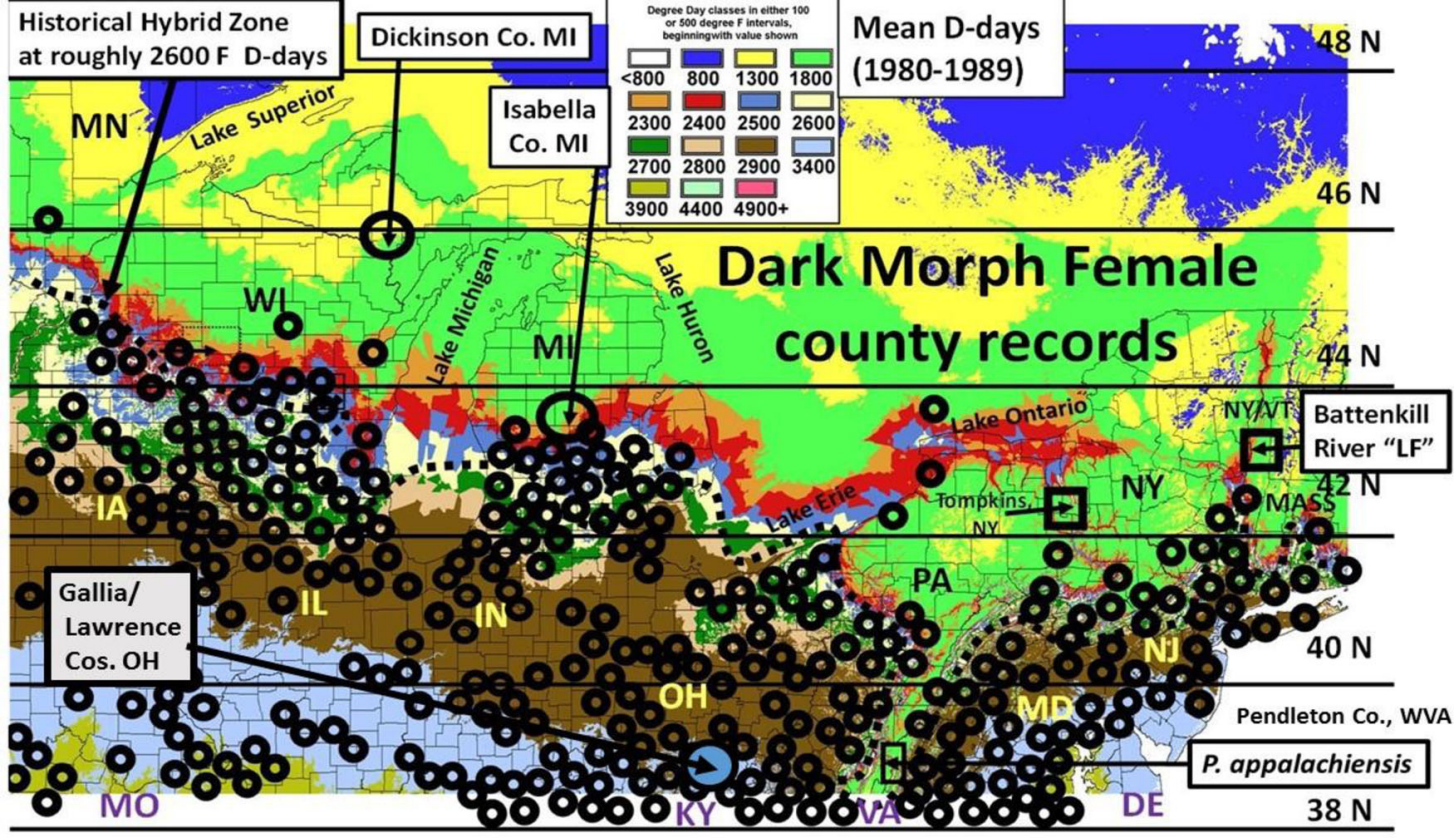
Distribution records for dark morph *P. glaucus* females across the Great Lakes region and New England overlaid on the mean thermal landscape (F degree‐days for the 1980–1989 decade; Scriber *et al*., 1996). Recent single dark morph records in Dickinson Co. in Michigan's UP and Isabella Co. in central Michigan are indicated, as are the “late flight” recombinant hybrid populations at Tompkins Co. NY and at the Battenkill River near the NY/VT border and the hybrid species, *P. appalachiensis*, at Spruce Mt., Pendleton Co., West Virginia (see Scriber *et al*., 2008). The northern limits to the dark morph females occurs where the *P. canadensis* species occurs (near the dotted line).

We have documented that the W‐linked dark allele (b+) does not occur in *P. canadensis* populations. The s+ allele for suppression of b+ appears at very high frequencies in *P. canadensis* populations while the Z‐linked s– “enabler” allele is in very high frequencies in *P. glaucus* populations (Scriber *et al*., 1996). The expression of wing color is controlled by epistatic interaction of genes on the Z‐ and W‐chromosomes in contrast to autosomal control of color patterns in other butterflies (e.g., *Heliconius*; Mallet, 1989). A long, but narrow, parapatric hybrid zone with *P. glaucus* occurs along the thermally delineated interspecific hybrid zone (from Minnesota and Wisconsin through Michigan, Pennsylvania, Vermont, New Hampshire, Maine, Massachusetts and most of New York State) at which the dark *glaucus* female frequency quickly goes to zero (Scriber *et al*., 1996; Fig. 1). Along this hybrid zone, introgression from *P. canadensis* might be responsible for the occurrence of fewer dark phenotypes than the W‐linked (b+) genotype might otherwise express (due to the Z‐linked suppressor, s+, or lack of enabler, s–; Scriber *et al*., 1987, 1996; Scriber, 2011). However, this low‐frequency suppressor (s+) could not account for the abrupt geographic decline in frequency of dark females by itself (see Scriber *et al*., 1996).

The factors determining the origin, spread and frequency of North American melanic (dark, mimetic) morph females of *Papilio glaucus* have fascinated naturalists since Linneaus described the two forms originally as different species *P. turnus*, and *P. glaucus* (see review by Pavulaan & Wright, 2002). For roughly 200 years the dark female morph has been known from Minnesota to Rhode Island, southward to Texas and central Florida, with dark female frequencies approaching 100% in the southern Appalachian Mountains, Alabama, and Mississippi (Edwards, 1884; Scudder, 1889; Mather, 1954; Scriber *et al*., 1996, 1998b). For example, in northern Georgia (Union and Fanning Counties), the dark morph females comprised 97% of all *P. glaucus* females reported during 1956–1959 (Brower & Brower, 1962). However, near the southern range limits of *P. glaucus*, near the tip of Florida, the frequency of dark morph females has also historically been at or near zero (Brower & Brower, 1962; Scriber *et al*., 1996; Lehnert *et al*., 2012).

The dark female (genotype b+, s–) of *P. glaucus* is a putative mimic of the distasteful *Aristolochia*‐feeding pipevine swallowtail, *Battus philenor* (L.) in the eastern half of the United States (Brower, 1958; Fordyce & Nice, 2003; Fordyce *et al*., 2005). The general correspondence in range limits of dark morph females with the geographical range of the putatively distasteful “model” (*Battus philenor*) was documented by Platt and Brower (1968) (see also Hagen, 1990; Scriber, 1996). A similar geographic range limit corresponding with the mimetic *Limenitis a. astyanax* (Fabr.) has also been described (Platt & Brower, 1968; Mullen *et al*., 2008). Decreased predation rates on mimetic butterflies such as dark morph females of *P. glaucus* that mimic the pipevine swallowtail, *Battus philenor* have been postulated based on indirect evidence or captive predator behavior studies (Brower, 1958; Codella & Lederhouse, 1989). However, rarely have they been investigated directly in the field (but see Sternberg *et al*., 1977). It has been suggested that the ventral (underside, while roosting) rather than the dorsal side of wings may be more exposed to predators, and it has been shown that male *P. polyxenes* are as effective mimics as females ventrally, but they are poorly protected dorsally (Codella & Lederhouse, 1989). The high roost mortality of *Papilio* early in the day and during inclement weather (when birds actively feed; Lederhouse *et al*., 1987) suggests that field tethering of dark and yellow morph females (or yellow males) would be an excellent way to experimentally evaluate differential local predation pressures directly.

However, despite the general assumptions that the mimics should be rare relative to the models, recent studies have shown that the advantage to mimetic butterflies is not frequency‐dependent on the model (which can be rare; Ries & Mullen, 2008). Also mimics often occur well outside the geographic range of the model (Pfennig & Mullen, 2010). In the *Papilio* mimicry complex, Brower and Brower (1962) report only a single *B. philenor* model in 1956 and 1959 in Highlands Co. Florida compared to more than 1000 females of *P. glaucus* counted during that time. Additionally, only a single individual of the *B. philenor* model species was seen in Highlands County during 1980–1986 (*n* = 166; Lederhouse & Scriber, 1987b).

The garden spider, *Argiope* spp. and the round orb‐weaver spider, *Nephila clavipes* L., are a major source of adult swallowtail butterfly mortality in Florida's Highlands County, and these spiders appear not to discriminate between dark and yellow morph *P. glaucus* (Scriber *et al*., 1998a). In fact, the general and nondiscriminatory impact of spider predation on these large *Papilio* species (*P. glaucus*, *P. troilus* L., and *P. palamedes* Drury) may be vastly underestimated (Uetz, 1992), as may be the case with lizards (Odendaal *et al*., 1987). Other potential reasons for differential effectiveness of mimicry in this system are discussed by Kunte (2009).

The natural selection frequency equilibrium for dark (mimetic) and yellow (tiger‐striped) *Papilio* females may depend on many factors, including types of predators and their learning capacities, the number and toxicity of model species (Kunte, 2009). Also, it was recommended that the sexual selection on different color morphs should be examined for direct measures of fitness impacts when possible, with focus on the mechanistic basis of male mate selection to determine if it is plastic, restrictive, or frequency‐dependent (Kunte, 2009). Such studies are described below.

Since *P. glaucus* males typically leave a single spermatophore that persists indefinitely, the number of matings and differences between yellow and dark females can be inferred (Burns, 1966). Higher spermatophore counts in wild yellow females had been proposed as a potential example of sexual preferences counteracting selection against these yellow (nonmimetic) females in areas with high frequencies of the *Battus philenor* models (Burns, 1966). However, subsequent studies of spermatophores have given conflicting results (Makielski, 1972; Platt *et al*., 1984; Lederhouse & Scriber, 1987a), suggesting that both female color morphs are mated at random, possibly due to similar ultraviolet wing colors of both yellow and dark female morphs (Platt *et al*., 1984). However, we have shown that neither the ultraviolet reflectance nor the blue coloration appear to be involved in male mating preferences (Aardema & Scriber, 2013), except that increased UV reflectance occurs in older, “worn” females (Aardema & Scriber, 2015).

Female age differences at time of capture could be a major factor accounting for this apparent randomness in mating frequencies as indicated by spermatophore counts (Lederhouse & Scriber, 1987a). However, in addition to age differences, other problems regarding the physiological or ecological significance of spermatophore numbers exist with spermatophore size differences, different proportions of apyrene and eupyrene sperm, nutrient status of males, etc. (Lederhouse *et al*., 1989, 1990; Stump & Scriber, 2006). Multiple mating in these *Papilio* species has been shown to be beneficial for restoring egg fertility/viability in older females (Lederhouse & Scriber, 1987a). However, many factors affecting the success or failure of these polygynous and polyandrous matings remain unknown (Lederhouse, 1995; Wiklund, 2003; Kunte, 2009), as is the role of sperm precedence (Stump & Scriber, 2006). To provide a more direct measure of male preference, female tethering in the field was conducted (see also Lederhouse, 1995; Deering & Scriber, 2002).

In general, the dark frequencies ranging locally from 30% to 80% have persisted (without much fluctuation locally from 1980 to 1997) across most of butterfly's range to the northern limits, near 40–42°N latitude within 50–150 miles from the historical hybrid zone with the univoltine *P. canadensis* in the Midwest (Fig. 1; Scriber *et al*., 1996). In the higher Appalachian Mountains in eastern United States, along the northern range limits of the multivoltine *P. glaucus*, the dark morph frequency drops precipitously to 0%–5% near the historical hybrid zone along the Great Lakes (Fig. 1) and into New England. This demarcation between voltinism and the limits of dark morph female records has historically been defined by areas with a total seasonal average degree‐days of less than 2600 F, base 50 °F ( = 1444 C, base 10 °C) (Luebke *et al*., 1988; Scriber *et al*., 2008; Scriber, 2011). In the recently described hybrid species, *P. appalachiensis* in the Appalachian Mountains (Scriber & Ording, 2005; Ording *et al*., 2010; Kunte *et al*., 2011; Zhang *et al*., 2013; Cong *et al*., 2015), the dark morph does occur inside the thermally defined hybrid zone in the mountains (Pavulaan & Wright, 2004; Scriber, 2011, 2014). It is primarily in hybrid zone areas with degree‐days accumulations of 2300–2800 F where introgression and recombination of X‐linked species‐diagnostic allele frequencies for Ldh and Pgd have been seen to diverge (making them diagnostic for the *P. appalachiensis* hybrid species on the warmer side of the hybrid zone, with Pgd allozymes fixed for *glaucus*‐like alleles and Ldh fixed for *canadensis*‐like alleles; Scriber & Ording, 2005; Scriber, 2011; Ryan *et al*., 2017, 2018b).

It has been shown that some of the univoltine, recombinant, late‐flying *P. appalachiensis* types on the warm side of the hybrid zone possess the Z‐linked enabler (s–) for melanism (Scriber & Ording, 2005; Scriber, 2011), and dark females can occur (see Pavulaan & Wright, 2004). Lab‐pairings have shown that the enabler (s–, from *P. glaucus*) has introgressed into some wild males of the 1999–2012 Vermont “Late flight” recombinant hybrids (and the central New York Tompkins Co. population; Hagen & Scriber, 1989; Fig. 1), but the W‐linked melanism gene (b+) itself has not yet introgressed into these hybrid populations (Scriber, 2011). The locations of all “type specimens” for *P. appalachiensis* reported in WVA, VA, MD, PA (see Pavulaan & Wright, 2002) and at the southern end of the Appalachian Mts. in northern GA all have thermal landscapes historically reflecting areas within the thermally defined hybrid zone (2700–2900 F degree‐days; Scriber, 2011).

The genetic basis of the female melanic trait in *P. glaucus* has been shown to be W (=Y)‐linked (females of butterflies are the heterogametic sex; Clarke & Sheppard, 1962; Scriber *et al*., 1996), with a Z (=X)‐linked modifier that enables (s–) or suppresses (s+) the expression of the W‐linked dark gene (b+ locus; Scriber *et al*., 1996) in the hemizygous females. Females that lack the black b+ allele or the s– enabler have the tiger‐striped (nonmimetic) yellow morph phenotype. The biochemical basis of the dopamine pathway of this melanic versus yellow (tiger‐striped) form has been worked out recently (Koch *et al*., 2000a,b; Ffrench‐Constant & Koch, 2003). The dark melanic gene on the W‐chromosome regulates the enzyme (DDC = dopa decarboxylase; see also Hodgetts & O'Keefe, 2006) that determines whether the yellow papiliochrome (using the enzyme beta‐alanyldopamine synthetase: BAS) or the dark melanic pathway will be followed in *P. glaucus* (Koch *et al*., 1998, 2000a,b). In the presence of both DDC and NBAD synthetase papiliochrome synthesis occurs, when the NBAD synthetase is absent, dopamine produced by DDC is shunted into the melanin pathway. Such fundamental enzymes such as DOPA decarboxylase and NBAD synthetase in *P. glaucus* may also play a general role in determining the “physiological costs” (pleiotrophic interactions) of insect melanism, as in *Drosophila* (True, 2003). Some potential costs of possessing the melanic capacity are evaluated here.

The “enabler” gene may involve the Z‐linked Tyrosine Hydroxylase (TyH: Putnam *et al*., 2007, and unpublished) or closely linked factors on the Z‐chromosome which are involved in production of dopa (which is common to both pathways: Koch *et al*., 2000a,b). This “enabler” may influence the late conversion of tyrosine into melanin that could result in incomplete penetrance in female color (intermediate “cinnamon” color, where yellow scales are brownish; Scriber *et al*., 2009a,b) due to differential interpretation of patterning signals in females (Ffrench‐Constant & Koch, 2003). This intermediate color has occurred only in daughters of dark morph females (i.e., with b+ Scriber *et al*., 1996) and may be induced by higher temperatures at the time of pupal metamorphoses (Ritland, 1986; Carpenter, 2014).

Genomic candidates for this melanin regulation have been identified by Cong *et al*. (2015). While 72% of the genome in the hybrid species, *P. appalachiensis*, is inherited from *P. canadensis* (including all four divergence hotspots involved in the circadian clock system regulating pupal diapause and adult eclosion; Cong *et al*., 2015), it has been shown that 6PGD (6‐glucophosphogluconate dehydrogenase) is closely linked with the melanism‐ enabling gene (s–) on the Z‐chromosome and inherited from *P. glaucus* (Hagen & Scriber, 1989). Possession and operating the Z‐linked “enabler” gene (s–) for the W‐linked melanism capacity (b+) appears very closely associated with two transcriptional factors near 6PGD (Cong *et al*., 2015) and may also have subtle pleiotrophic effects or physiological/ecological “costs.” Some of the most obvious developmental and survival traits in *P. glaucus* that might be differentially impacted by melanism were experimentally examined here for dark morphs and yellow morphs and their offspring.

## Materials and Methods

### Calculation of local growing degree‐days and seasonal thermal landscape mapping

The daily thermal unit accumulations through the growing season Mar 1 to Oct 31 (in Fahrenheit degree‐days above a base 50° = 9/5 C degree‐days above a base 10 °C) were calculated for each of more than 2000 stations in northeastern United States (by Zedex, Inc, Bellfonte, PA, USA). The base developmental threshold was calculated as the inverse of the time required to complete development when plotted against rearing temperatures (Scriber & Lederhouse, 1983). The historical degree‐day accumulations were run in a GIS spatial program with interpolation to 1 km² and presented geographically using colors to indicate different isotherms at 100 °F intervals between 2300 and 2900 (and at 500 °F intervals to the south and the north of these critical thermal transition zones). Thermal landscapes for the individual years and the 10‐year average accumulations were prepared for use here. Other climatic data were obtained from the USDA Plant Hardiness Zone maps and the Climatic Atlas of Michigan (Eichenlaub *et al*., 1990). Historical annual degree‐day accumulations for Athens, Georgia (from 1960 to 2017; Fig. 8) were obtained from the North Carolina State website (climate.ncsu.edu).

### Distribution records for dark morph females

**Figure 2:**
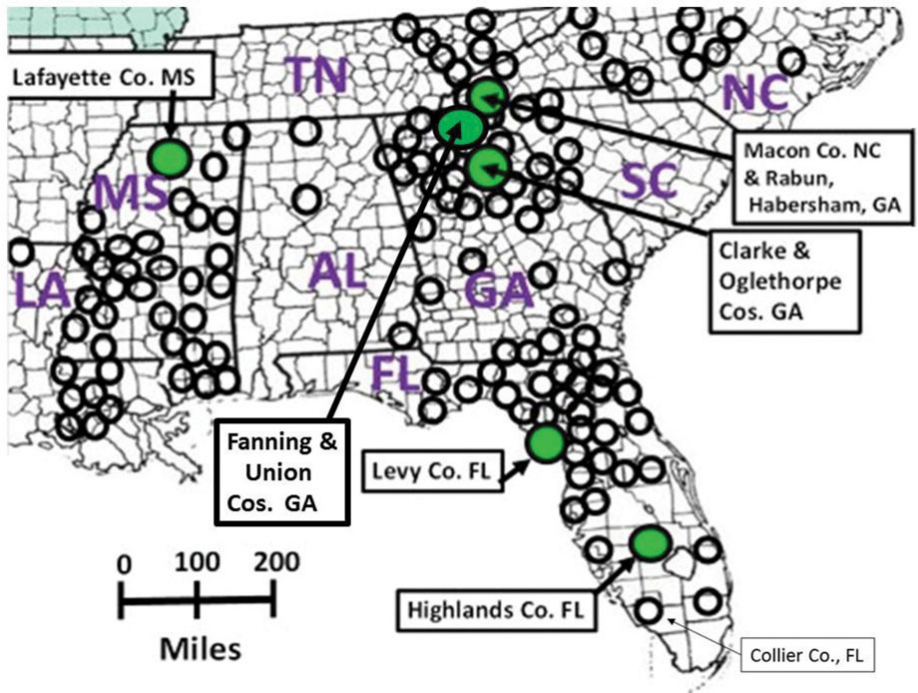
Distribution (southeastern United States) of dark morph *P. glaucus* females with key sampling sites indicated. There are an additional 432 counties from the 9 states shown here where documented records of *P. glaucus* exist. Unfortunately, these do not indicate whether or where dark morph femeles occur [see North American Butterflies and Moths list (abirdshome.com)].

Dark morph females of *P. glaucus* (and *Battus philenor*) were examined for county of capture records at many University, State, Government and Public Museums (many listed earlier in Scriber *et al*., 1996). These records were supplemented by range records published in Ebner, 1970; Irwin & Downey, 1973; Shapiro, 1974; Opler & Krizek, 1984; Shull, 1987; Iftner *et al*., 1992; Allen, 1997; Layberry *et al*., 1998; Nielsen, 1999; and the R. Poole Nearctica website (Butterflies and skippers of North America). In addition, our lab (and various collaborators) have made extensive and continuing collections across eastern North America for 35–40 years. We include our own recent and previous records here (since 1996, including the post‐1998 warming climate impacts; Fig. 1). Also included for the first time are the Massachusetts Statewide Butterfly survey of 186 State‐wide sampling quadrats during 1986–1990. Many counties without records of dark morph females in the central regions (e.g., OH, KY, TN, AK, LA, MS, AL, NC, SC, GA) may contain dark females, but have just not been documented in the literature or research collections (Fig. 2; see Mather, 1954). Unfortunately the recent online county records for many states do not state whether dark morph females occur there, just listing *P. glaucus*. For example, 432 counties from the 9 states shown in Fig. 2 have been documented for *P. glaucus* presence (but without any differentiation of dark or yellow morph females). In contrast, at the northern edge of the range where dark females have been rare or nonexistent, very intense and sustained sampling has been made by our group and others (in WI, MI, NY, VT, MA, CT, NH, ME, and Canada). In fact, it is almost certain that most collectors in these regions look even more intensely for “rare” specimens, such as dark females (e.g., Scriber *et al*., 1998a,b; Nielsen, 1999). Field‐collected and lab‐reared individuals were measured for various morphometric traits, including forewing lengths (Table 1; see also Lehnert *et al*., 2012; Scriber, 2014).

**Table 1:** Size (forewing lengths; mm ± SD) of yellow morph and dark morph females of *Papilio glaucus* from different locations in eastern North America. (Data are presented as a mean ± SD). Populations include: Gallia & Lawrence Cos. OH; Rabun & Habersham Cos (GA) with adjacent Macon Co. NC; Clarke & Oglethorpe Cos. GA; Levy Co. FL and Highlands Co. FL. Note that individuals in the early flights (first generation in March or April) are smaller the those of the late season

Location	Year	*n*	Yellow females	*n*	Dark females
Ohio (Lawrence & Gallia Cos.; 38.5°N lat.)					
	1988 J‐Aug	12	54.3 ± 1.8	90	54.9 ± 2.6
	1989 J‐Aug	25	57.0 ± 2.6	123	57.1 ± 2.5
	1991 J‐Aug	11	57.4 ± 2.1	52	57.9 ± 2.2
	1992 J‐Aug	28	55.7 ± 2.7	123	56.9 ± 2.6
	1996 J‐Aug	40	57.7 ± 2.9	46	58.2 ± 2.3
	2008 J‐Aug	6	54.3 ± 1.4	9	56.2 ± 2.7
Rabun, Habersham, Macon (nGA & NC; 34.9°N)					
	2008 Aug	32	58.2 ± 1.8	18	58.0 ± 2.6
	2009 Aug	25	57.3 ± 2.6	19	58.0 ± 3.0
Georgia (Clarke & Oglethorpe Cos.; 34.0°N)					
	1988 Aug	34	58.7 ± 2.7	64	59.4 ± 3.3
	1989 Aug	15	59.5 ± 2.6	44	58.7 ± 3.1
	1993 Aug	57	57.8 ± 2.8	166	58.2 ± 2.2
	1994 Aug	52	58.4 ± 3.5	105	57.9 ± 3.0
	1995 Aug	56	59.4 ± 2.9	152	59.1 ± 2.8
	2008 Aug	23	59.0 ± 2.0	22	59.1 ± 2.1
	2009 Aug	21	58.3 ± 1.8	16	59.5 ± 2.9
	1989 M‐Apr	43	52.0 ± 2.3	92	51.7 ± 3.6
Florida (Levy Co.; 29.1°N)					
	2000 Apr	46	64.9 ± 2.6	73	65.5 ± 2.6
	2003 M‐Apr	6	62.5 ± 3.8	4	63.8 ± 1.0
	2003 Sept.	8	68.6 ± 3.2	3	68.7 ± 2.9
	2004 M‐Apr	14	64.4 ± 3.1	20	64.1 ± 1.9
	2006 M‐Apr	56	62.9 ± 3.1	75	63.1 ± 2.8
	2008 M‐Apr	132	64.2 ± 2.3	166	64.8 ± 2.5
Florida (Highlands Co. 27.3°N)					
	1988 M‐Apr	36	62.4 ± 3.3	24	62.3 ± 3.3
	1994 M‐Apr	37	62.2 ± 3.6	18	62.8 ± 2.5
	1995 M‐Apr	14	61.7 ± 1.6	3	61.3 ± 1.5
	1995 Sept.	6	66.3 ± 2.6	8	68.1 ± 1.7

No significant differences were observed for dark versus yellow morph females at any time/location. For both color morphs the early flight forewings (March to April) are smaller than those of late summer (see also Scriber *et al*., 2014).

**Figure 3:**
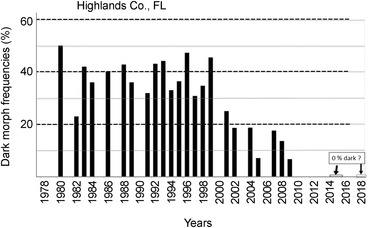
Annual (1980–2018) dark morph frequencies in Highlands Co. Florida (27.3°N latitude). Major drainage and habitat alterations in 1959 and resulted in drastic population reductions (see Lederhouse & Scriber, 1987b). Continued declines in populations have steadily occurred in the Parker Islands, the Lake Placid, Lake Istokpoga, as well as the Archbold Biological Station area of Highlands Co., FL (which have become locally extinct, or on the verge; only 3 yellow females were seen in 2015, and only 2 yellow females in 2018). Annual sample sizes from 1980–2009: 14, 40, 12, 45, 13, 60, 311, 111, 109, 156, 11, 44, 19, 132, 44, 12, 60, 16, 42, 17, 44, 15; total 1494 females.

**Figure 4:**
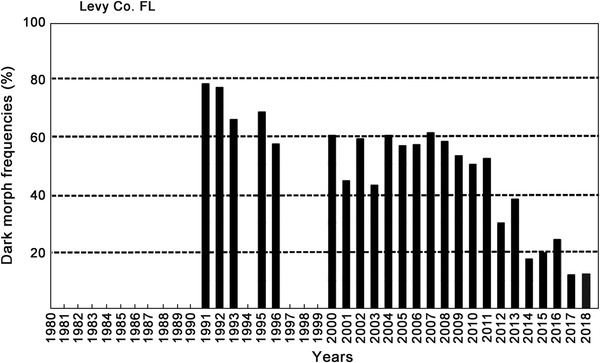
Annual dark morph frequencies in Levy Co. Florida (29.1°N lat). Annual sample sizes from 1991–2018: 18, 17, 26, 50, 14, 135, 18, 254, 14, 45, 55, 132, 133, 729, 83, 40, 25, 67, 97, 40, 153, 182, 175, 120; total 2652 females.

**Figure 5:**
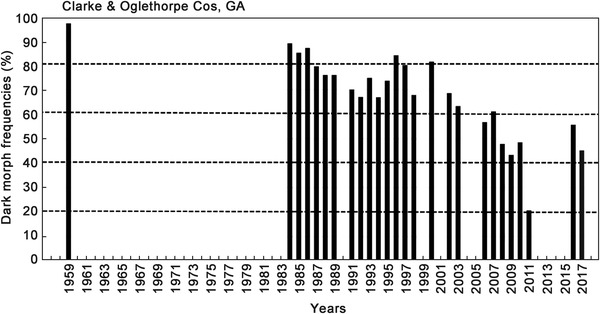
Annual dark morph frequencies in Clarke & Oglethorp Cos, in north central Georgia (34.0°N lat.). The 1959 data from adjacent Fanning and Union Cos. Georgia (see Fig. 2) are from Brower and Brower (1962). Annual sample sizes from 1984–2017: 33, 42, 61, 99, 290, 248, 188, 208, 42, 222, 157, 208, 46, 85, 149, 43, 105, 70, 60, 18, 76, 37, 33, 29, 100, 112; total 2761 females.

**Figure 6:**
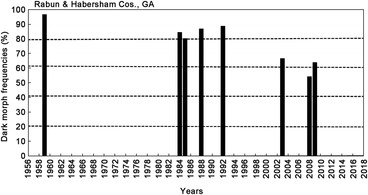
Annual dark morph frequencies in Rabun & Habersham Cos. northernmost Georgia and adjacent Macon Co. in western North Carolina (34.9 °N lat.). The 1959 data are from Brower and Brower (1962). Annual sample sizes from 1959–2009: m33, 13, 66, 92, 27, 6, 46, 50; total 333 females.

**Figure 7:**
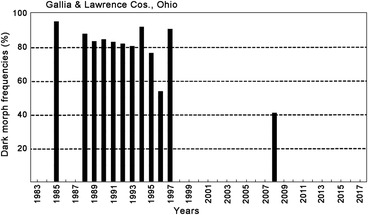
Annual dark morph frequencies in Gallia & Lawrence Cos. in southeastern Ohio (40.4°N lat.). Annual sample sizes from 1985–2008; 38, 102, 158, 87, 49, 255, 25, 23, 108, 86, 30, 22; total 983 females.

The key populations sampled for multiple decades for change in frequencies of dark females include; Highlands Co. FL (lat 27.3°N), Levy Co. FL lat. 29.1°N) Clarke and Oglethorpe Cos. in northern GA (lat 34.0°N); Rabun/Habersham Cos. GA (lat. 34.0°N), and Gallia & Lawrence Cos. OH (lat. 38.5°N). The total number of females sampled for these locations were: *n = *1494 for Highlands Co. FL (1980–2018); *n = *2652 for Levy Co. FL; *n = *2761 for Clarke and Oglethorpe Co. GA; *n = *333 for Rabun and Habersham Cos. GA and Macon Co. NC; and *n = *983 for Gallia and Lawrence Cos. OH. Specific annual sample sizes are indicated in the captions of Figs. 3, 4, 5, 6, 7.

### Oviposition and larval rearing

Field‐collected (or hand‐paired) females were individually placed in multi‐choice oviposition arenas where they could choose individual leaves as the arenas rotated in front of a bank of 60 W lights (once each 6 min; see Scriber, 1993; Mercader & Scriber, 2005, 2007). Eggs were collected daily and counted according to host plant species and then moved into plastic dishes in a controlled environment chamber. Females were fed a 20% honey water solution daily and returned to the arena for another day of oviposition. Egg viability was determined by comparing the proportion of hatching eggs of the total eggs produced by each individual female (Table 2). Means of the families were also compared for yellow and dark *P. glaucus* mothers from MI, WI, OH, IN, MO, VA, GA, FL, and for primary hybrids of dark and yellow females mated to *P. canadensis* males (Table 2).

**Table 2:** Mean egg viability for families (*n*) of yellow and dark morph *Papilio glaucus* (Pg) from multiyear populations across the eastern United States. Data are expressed as a mean ± SE of the individual family means (proportion of neonate larvae of the total eggs produced) for each location. No significant differences between color morphs were observed

Population	*n*	Yellow mothers	*n*	Dark mothers
MI (St. Joseph Co.)	19	61.1 ± 6.0	6	70.5 ± 12.0
WI (Dane Co.)	3	66.6 ± 16.6	2	75.1 ± 8.7
OH (Lawrence Co.)	32	71.5 ± 3.6	43	72.7 ± 3.4
IN (Vigo Co.)	8	65.4 ± 7.1	12	72.5 ± 7.0
MO (St. Charles & Lincoln Cos.)	4	74.0 ± 9.9	22	80.6 ± 3.9
VA (Nelson Co.)	11	70.2 ± 2.3	15	69.8 ± 4.3
GA (Clarke Co.)	29	62.7 ± 5.6	13	69.5 ± 5.9
FL (Highlands Co.)	12	48.2 ± 7.9	3	40.3 ± 20.5
Primary hybrids (g × c)	16	45.1 ± 8.4	34	60.0 ± 5.4

In primary hybrids, the Pg mothers (virgin yellow or dark) are listed first, with the *P. canadensis* (Pc). fathers last. The sample years for these populations were: MI (1997, 1999, 2000, 2003); OH (1995, 1996, 1997); IN (2004); MO (1998, 1999); VA (2000); GA (1997, 1998, 1999, 2000); FL (1995); hybrids (2001, 2002, 2003, 2004). The average number of eggs/female for different populations (mean of family means for fecundity) ranged from 64 to 199 per population. As with the egg “viability” percentages above, the “fecundity” differences (total egg production; not shown) between yellow and dark morphs were also not significant for any population or hybrids (*t*‐tests; *P* = 0.10) in any case.

Field‐reared *P. glaucus* pupae reared from various dark and yellow mothers were stored for the winter at 3–5 °C and brought out after 5 months and randomly assigned to seven thermal regimes all at long‐day 18 : 6 photophase for adult emergences at 11, 14, 17, 20, 23, 26, and 29 °C. (Table 3 and Fig. 9). Some larvae were reared to pupation. Pupae were weighed 3–4 d after the prepupa formed and maintained at long‐day conditions (18 : 6 h photoperiod) for 5 weeks. This period permitted us to determine the capacity for direct development to occur (od‐gene). After 6 weeks, all pupae were placed and maintained for the “winter” in 3.5– 4.0 °C total darkness for 4–6 months as were field‐reared pupae.

**Table 3:** Postdiapause pupal eclosion time (days) of *P. glaucus* adults (from either dark or yellow morph mothers) under seven different postdiapause temperature regimes (all at 18 : 6 photoperiod). Pupae were all field‐reared in Pennsylvania (by Wm. Houtz) and stored for the winter in darkness at 3–5 °C in our lab, until brought out after overwintering for 6 months for adult emergence in controlled environment chambers the following spring. Data are presented as a mean ± SE (*n* = individuals)

Temperature	(*n*) Males (from DK)	(*n*) Males (from YL)	(*n*) Females (from Dark)	(*n*) Females (from Yell)
11 °C	(8) 195.6 ± 23.3	(8) 192.3 ± 23.4	(4) 244.8 ± 29.1	(5) 214.0 ± 32.8
14 °C	(31) 65.4 ± 1.9	(27) 66.3 ± 4.5	(27) 73.9 ± 2.6	(18) 73.9 ± 6.5
17 °C	(40) 34.1 ± 0.8	(39) 29.3 ± 0.8*	(36) 36.4 ± 0.9	(36) 32.4 ± 1.0*
20 °C	(55) 23.5 ± 0.7	(71) 26.6 ± 1.3	(64) 27.1 ± 1.0	(55) 27.3 ± 1.0
23 °C	(33)19.1 ± 0.6	(45) 19.2 ± 0.6	(49) 21.1 ± 0.7	(28) 19.6 ± 0.7
26 °C	(49) 15.4 ± 0.5	(65) 14.1 ± 0.3	(48) 16.3 ± 0.7	(51) 14.3 ± 0.5
29 °C	(38) 12.0 ± 0.5	(33) 12.1 ± 0.8	(27) 12.1 ± 0.6	(33) 17.1 ± 1.7*

*Significant differences between color morphs within gender class (*P *= 0.05, Tukey's *t*‐test).

The seven‐choice oviposition arenas (Scriber & Gage, 1995; Scriber, 2002b, 2010) included tulip tree (*Liriodendron tulipifera* L.), hop tree (*Ptelea trifoliate* L.), white ash (*Fraxinus americana* L.), black cherry (*Prunus serotine* Ehrh.), spicebush (*Lindera benzoin* L. Blume), *Rhamnus* spp. and quaking aspen (*Populus tremuloides* Michx.). Data are expressed as family means for dark and yellow females from several locations and/or years (including Raleigh, NC; Clarke Co. GA; Gallia and Lawrence Counties, OH; and Lancaster Co. PA; Table 4).

**Table 4:** Seven‐choice oviposition preferences means of individual dark and yellow females (*n*). The total egg production (fecundity) could not be compared since females were of different ages at capture with unknown number of matings (which could affect fecundity and fertility)

State (Co.)	*n*	TT	HT	WA	BC	SP	RH	QA	EGGS
**NC, Raleigh**									
Dark	11	32.9	20.4	18.2	12.8	13.5	0.7	1.4	66.8
Yellow	6	38.6	23.4	11.0	14.9	8.8	2.3	1.1	85.2
**GA Clarke Co.(early Aug)**									
1995 Dark	13	47.6	19.7	10.9	10.6	9.0	0.4	1.7	61.2
1995 Yellow	12	44.0	19.2	10.1	13.8	9.2	2.6	1.2	51.6
1996 Dark	16	41.3	14.7	23.5	4.6	10.3	0.7	4.1	61.4
1996 Yellow	22	38.6	12.6	33.2	7.8	13.8	1.4	3.1	95.7
**GA Clarke (late Aug)**									
1995 Dark	49	40.4	16.9	13.3	13.4	11.1	1.8	3.0	69.6
1995 Yellow	27	37.7	21.1	15.6	11.2	7.2	1.4	4.2	78.3
**Ohio (Gallia Co.)**									
1995 Dark	39	32.7	19.0	18.1	17.0	10.3	1.4	4.2	73.7
1995 Yellow	7	33.7	17.9	17.7	12.4	15.0	0.0	3.3	52.6
**Ohio (Lawrence Co.)**									
1995 Dark	16	35.0	21.2	14.5	13.0	9.5	1.7	5.1	86.6
1995 Yellow	5	36.0	22.0	14.9	14.3	7.7	1.2	2.8	70.6
1996 Dark	9	42.7	19.5	14.8	8.7	12.4	1.0	0.9	139.9
1996 Yellow	14	43.1	25.4	9.4	6.5	9.0	2.4	4.2	141.2
**Pennsylvania (Lancaster Co.)**									
1993 Dark	7	34.2	23.5	19.3	5.9	8.3	4.8	4.0	61.4
1993 Yellow	5	38.6	16.7	24.7	7.7	5.5	3.8	3.1	50.8

The seven‐choice assays contained: TT = tulip tree (*Liriodendron tulipifera*, Magnoliaceae), HT = hop tree (*Ptelea trifoliata*, Rutaceae), WA = white ash (*Fraxinus american*a, Oleaceae), BC = black cherry (*Prunus serotina*, Rosaceae), SP = spicebush (*Lindera benzoin*, Lauraceae), RH = *Rhamnus* spp (Rhamnaceae), QA = quaking aspen (*Populus tremuloides*, Salicaceae). In all cases the hierarchy of preferences was nearly identical for dark and yellow females for any year or location.

Neonate larvae were randomly and gently transferred (using fine hair brushes) onto different host plant species for survival and growth bioassays. Subsequent larval rearing to pupation was in controlled environment chambers (photoperiods of 18 : 6 h, or short day 12 : 12 h, at selected temperatures). Neonate larvae were set up in a split‐brood design on tulip tree, black cherry, and quaking aspen leaves in groups of 1–3 per dish. Data are expressed as the mean of the families of dark versus yellow from each of two locations (Nelson Co. VA in 2000 and Lincoln Co. MO in 1999; Table 5). In addition, the neonate survival of primary hybrids using dark and yellow females mated to *P. canadensis* were included for potential differences in broods of different colored moms. Total durations from egg to pupation under summer long‐day (16 : 8 h) conditions were compared for families from 21 dark and 18 yellow Dane Co. Wisconsin mothers (Table 6).

**Table 5:** Neonate larval survival of dark and yellow morph offspring on three key host plant species (TT = tulip tree, Magnoliaceae; BC = black cherry, Rosaceae; QA = quaking aspen, Salicaceae). Data are presented as a mean of family means ± SD (*n* = families)

Population	*n*	TT	*n*	BC	*n*	QA
Virginia (Nelson Co.)						
Dark	14	74.2 ± 18.8	11	78.7 ± 21.4	8	4.3 ± 8.8
Yellow	11	77.7 ± 21.6	9	84.6 ± 8.3	11	6.3 ± 5.4
Missouri (Lincoln Co)						
Dark	14	78.5 ± 19.0	14	87.3 ± 12.6	14	2.4 ± 6.9
Yellow	3	72.1 ± 4.1	2	95.5 ± 5.5	3	0.0 ± 0.0
Hybrids						
PA (Dark) × Pc	4	88.5 ± 5.7	4	85.2 ± 11.8	4	86.0 ± 5.2
PA (Yellow) × Pc	15	75.0 ± 16.9	15	72.6 ± 24.6	15	72.0 ± 21.0

No significant differences between means for dark and yellow at different locations (*t*‐test; *P *= 0.05).

**Table 6:** Larval durations from neonates to pupation at different temperatures (16 : 8 photo : scotophase) of dark and yellow female offspring. Data presented as a mean ± SE

	28 °C		25 °C		22 °C		19 °C	
Population	(*n*) mean ± SE		(*n*) mean ± SE		(*n*) mean ± SE		(*n*) mean ± SE	
Wisconsin (Dane Co.)								
Dark	(21) 21.5 ± 0.6		(24) 28.2 ± 0.8		(8) 32.1 ±1.1		(10) 57.4 ± 1.1	
Yellow	(18) 21.2 ± 0.4		(14) 26.9 ± 0.5		(15) 34.1 ± 0.9		(11) 54.7 ± 1.1	

None of the dark/yellow differences are significant (*P* = 0.05; *t*‐tests).

Short term “winter stress” conditions were imposed upon pupae of dark and yellow families derived from Lancaster Co. PA and Clarke Co. GA). These conditions included 4 d in mid‐January of cold stress at –21, –18, or –13 °C. In addition a 4‐d winter warming period of 20 °C for mid‐January was included for comparison with control pupae allowed to remain at 4 °C for the entire winter (Table 7). Stressed pupae were returned to the 4 °C chambers after the 4‐d stress periods (see also Scriber *et al*., 2012).

**Table 7:** Diapausing pupal mortality in the spring after 4 d of exposure during mid‐winter diapause to four different short term mid‐winter temperature stresses (in January). Pupae were immediately returned to winter storage at 4 °C (the control) after the imposition of four stress treatments, and all diapausers were later removed and individually set up at 22 °C 18 : 6 photoperiod for adult emergences in mid‐April

	–21 °C	–18 °C	–13 °C	+20 °C	Controls (4 °C)
Temps.	(*n*) %	(*n*) %	(*n*) %	(*n*) %	(*n*) %
PA dark	(20) 0	(2) 0	(20) 0	(20) 0	(9) 0
PA yellow	(18) 16	(7) 0	(26) 0	(21) 0	(16) 0
GA dark	(5) 80	(22) 27	(5) 0	(5) 0	(13) 0
GA yellow	(5) 60	(6) 17	(5) 0	(5) 0	(8) 0

While Georgia and Pennsylvania populations at –21 °C differed in winter stress susceptibility, the dark and yellow pupal offspring of PA and GA did not.

Pupae of Pennsylvania reared dark and yellow morph females were put in the usual diapause storage conditions at our lab (4 °C in darkness) and weighed at mid‐winter (December). Live weights of all pupae were taken on a macroanalytical balance (and a subsample was taken to determine water content by deep freezing and subsequent drying). After 6 months the pupae were removed from diapause conditions weighed live and sacrificed for dry weight determinations (Table 8).

**Table 8:** The weight losses and percent body water of diapausing pupae (Pennsylvania‐reared *P. glaucus*) at mid‐winter (December, with subsamples for water content) and in the spring (late April) after 6 months of winter diapause at 4 °C in darkness. Data are expressed as a mean ± SD

Time spent in diapause (at 4 °C, in darkness)	*n*	Water content (mid‐winter) (%)	Weights (mid‐winter) (mg)	*n*	Live weight loss (%)	Water content (spring) (%)
*P. glaucus* (dark mothers)	12	72.8 ± 1.5	1236.9 ± 149.7	12	9.8 ± 2.6	72.7 ± 2.5
*P. glaucus* (yellow mothers)	12	73.4 ± 4.1	1105.9 ± 106.7	12	10.6 ± 2.4	73.9 ± 1.7

No significant differences between the dark morph and yellow morph *Papilio* means are indicated (*P *= 0.05; Tukey's tests).

Severe mid‐winter conditions were simulated by exposing diapausing pupae of dark and yellow morph females to 10‐, 20‐, 35‐d durations at –18 °C (in the middle of their normal diapause conditions at 4 °C darkness) compared to the control group (undisturbed diapausers). Spring emergences were allowed after 6 months and the times for development into eclosing adults from pupae were determined (Table 9).

**Table 9:** Impacts of extended winter cold stresses (10 d, 20 d, 35 d at –18 °C; and normal, undisturbed controls = 0 d) on dark and yellow morph *P. glaucus* offspring at mid‐winter during their postdiapause development and adult eclosion from pupal diapause

	From dark morph mothers		From yellow morph mothers	
Mid‐winter stress duration	(*n*) Sons	(*n*) Daughters [dead]	(*n*) Sons	(*n*) Daughters [dead]
35 d	(9) 20.4 ± 0.7	(5) 21.0 ± 3.7 [6]	(9) 21.9 ± 3.8	(8) 23.5 ± 5.2 [3]
20 d	(9) 18.8 ± 2.0	(8) 20.1 ± 4.3 [2]	(12) 19.5 ± 3.5	(4) 21.8 ± 2.5 [2]
10 d	(7) 17.4 ± 1.5	(12) 18.3 ± 1.9 [1]	(12) 19.2 ± 3.7	(7) 19.5 ± 2.2 [1]
0 d (control)	(8) 17.4 ± 1.6	(12) 18.3 ± 2.1 [0]	(6) 17.7 ± 4.2	(13) 21.2 ± 2.9 [0]

No significant differences occurred in postdiapause development and time to adult eclosion between sons or daughters of dark or yellow mothers (*t*‐tests, *P* = 0.05).

### Tethering for male mate preferences for (and differential predation of) yellow and dark females

Based on spermatophore counts in wild female *P. glaucus* of both color morphs, it had previously been proposed that the preference of wild males for the yellow (nonmimetic) morphs might negate the higher bird predation on the yellow morph, preventing the dark morph from going to fixation where the *B. philenor* model was most abundant (Brower & Brower, 1962; Burns, 1966; but see Pliske, 1972, 1973; Platt *et al*., 1984; Tables 10–12). A more direct measure of sexual preferences has been made using size‐matched, virgin, paired tethering in key field locations (see below).

**Table 10:** Field mating preferences of males for tethered, size‐matched, virgin females (dark and yellow morphs in a 2‐choice array, with methods as in Deering & Scriber 2002). Local population frequencies of dark morph females previously and during the study are indicated (2001, Isabella, MI; 1989 & 1990 Highlands, FL; 2003 and 2008 Levy, FL; and 1989 Lawrence/Gallia, OH; see also Figs. 3, 4, 5, 6, 7; Scriber *et al*., 1996, 1998). The range of annual means of dark frequencies has declined significantly in recent years for the southern populations, and may result in a reduced preference for dark morphs if frequency‐dependent mating behavior persists in future studies (e.g., Ohio is now only 50%–60% dark, Levy, FL is only 12%–24% dark; and Highlands is 0–15% dark; Figs. 3, 4, 5, 6, 7)

Location (latitude)	Local freq.^*^	Mating preferences (copulations)		% dark pref.
Isabella Co. MI (43.4°N)	0 %	2 dark	43 yellow	4.6%
Highlands Co. FL (27.3°N)	30%–45 %	23 dark	66 yellow	28.6%
Levy Co. FL (29.1°N)	45 %–60 %	7 dark	5 yellow (2003)	58.3%
		5 dark	5 yellow (2008)	50.0%
Lawrence/Gallia				
Cos. (Southern Ohio; 40.1°N)	75%–85 %	37 dark	12 yellow	75.5%

Isabella (JM Scriber and H Hereau, unpubl.); Levy Co. FL (JMS unpubl. 2 different years); Highlands Co. FL and Ohio (RC Lederhouse and JM Scriber, unpubl.data). Ohio males are three times more likely to mate with black than yellow (*χ* ^2^ = 12.7, *P* < 0.001) while Highlands Co. Florida males are three times more likely to mate with yellow than black females (*χ* ^2^ = 14.4, *P* < 0.001).

Since *P. canadensis* and *P. glaucus* mating systems involve patrolling males (Brower, 1959; Lederhouse, 1995), the male mating preferences in the field have been directly assessed by using size‐matched, virgin females on experimental tethers to evaluate prezygotic isolating mechanism. Female refusals are rare, but easily detected and recorded. Different individual females were used to avoid pseudo‐replication (see Deering & Scriber, 2002) and relative positions of tethered females have been switched at 20‐min intervals for all 2‐h study pairs. We have had 285 field copulations of *P. canadensis* males in a single 4‐h afternoon at one site in northern Michigan (Deering & Scriber, 2002), and thus, rigorous replication of behavioral response traits was feasible. In studies of field tethered virgin, size‐matched yellow and dark female *P. glaucus* females, we noticed that *P. canadensis* males didn't seem to even recognize the dark morphs, but did mate with yellow morph females. In fact, when offered yellow *P. glaucus* with their own monomorphic yellow *P. canadensis* females, *P. canadensis* males overwhelmingly preferred yellow *P. glaucus* females (82% of all field copulations; *n = *476; Deering & Scriber, 2002). These interspecific mating results led us to re‐examine intraspecific sexual selection by males (of yellow or dark females of *P. glaucus*) as one factor influencing the distribution/frequency of dark morph *P. glaucus* in areas where dark female frequency declines. Sites examined for differential mate preferences included Isabella Co, MI, Lawrence/Gallia Cos. OH, Highlands and Levy Cos. FL where the natural frequencies of dark morphs ranging from 0% to 90 % (Table 10). Note that the recent dark morph female frequencies at these Florida (Highlands and Levy counties) and Ohio (Gallia and Lawrence counties) sites have decreased since the tethering studies were done (see Figs. 3, 4, 5, 6, 7).

In preliminary Florida and Michigan predation studies, dark and yellow females were similarly tethered on 0.5 m long thin black threads with alligator clips at one end for attachment to branches in the field (as used successfully for tethered mating preference studies in MI and FL; Deering & Scriber, 2002). The roosting site (e.g., upper or under sides of leaves) was chosen by females, since they could fly on the tether. At each field location and at 10–20 m intervals, 5–6 pairs of yellow and dark females (each pair separated by 1.5–2.0 m) were checked in late morning to provide an index of any differential localized predation differences.

### Hybridization

Interspecific hybrids were produced by hand‐pairing of virgin (lab‐reared) females with field‐captured or lab males. The offspring of dark and yellow mothers paired with males of *P. canadensis*, *P. rutulus*, or *P. eurymedon* were reared and examined for sex‐ratio differences between the families of the different color morphs due to genetic incompatibilities as possible Haldane Effects (Tables 13 and 14). The W‐linked melanic gene b+ was examined to see if it might be the cause of (or associated directly with) pupal inviability in such hybrid female offspring derived from melanic females. The sex of dead pupae was determined using methods described in Carter and Feeny (1985).

A very informative laboratory backcross family (#18006) was created by pairing a dark morph female and a hybrid (g × c) father. The mother produced 388 eggs, 286 neonate larvae, and 112 pupae (many larvae died in host suitability assessments). Of these pupae 20 females (17 dark and intermediate and 3 yellow) and 39 male adults developed and emerged under long‐day (18 : 6 h) lab conditions. Four males and four females (all dark; s–, od+) emerged in year 2, and one male and four females emerged in year 3 (all dark females, s–, od+ and mixed for Ldh and Pgd allozymes). Many recombinations of diagnostic traits were observed in these offspring (below), but one in particular is that, of the 20 direct developing (nondiapause, od–) females, 19 had the Ldh‐100 allele (showing close linkage between od– and Ldh‐100). Of these 20 females, 17 were dark or intermediate (s–, with b+; Scriber, 2011). In addition, genetic incompatibility in female pupae (depending on the Z‐linked “canadensis‐type” Ldh and Pgd alleles) was assessed as for other backcrosses (Hagen & Scriber, 1995; Table 15).

## Results

### Updated distribution records for dark morph females (since regional climate warming 1997–2018)

The geographic range limits of the dark morph females of the Eastern Tiger Swallowtail Butterfly, *P. glaucus*, across eastern North America have remained basically unchanged over the past several decades from Canada to eastern Texas and southern Florida, and, in addition, the local frequencies had remained basically stable during the period up to 1997 (Scriber *et al*., 1996). However, at the northern edge of the species range, recent observations in Michigan report the occurrence of isolated dark morphs. In 1997, a dark morph female was caught in Dickinson County in the Upper Peninsula of Michigan (well north of the hybrid zone; Fig. 1) and was likely to represent a “blow‐in” from strong winds from the south a few days earlier (Scriber *et al*., 1998b). In 2000, a dark morph female was captured by Ted Herig in central Michigan, just at the northern edge of the hybrid zone, where no dark females had ever previously been reported (Neilsen, 1999; Blomberg & Herig, 2014; Fig. 1).

Near the southern edge of the dark morph distribution records, dark females were documented for the first time in southwestern Florida, in Collier Co., Martin Co., and Sarasota Co. (Ken Werner, Dave Baggett, Bill & Debi Hill; pers. comm.). Previously, only yellow morph females had been reported south of Highlands Co. Florida (Brower & Brower, 1962; Scriber *et al*., 1996; Fig. 2). However, no dark morph records exist to date for nearby Munroe, Dade, Broward, Palm Beach, Charlotte, Glades, Lee, or Hendry Counties in the southern tip of Florida (Fig. 2). Since 1996, we have also collected dark females from other more northern Florida counties for which no known published records were previously known, including Taylor, Baker, Clinch, Ware, Charlton and Jefferson Counties. James Maudsley (pers. comm.) has also collected dark females in Columbia Co. Florida. A few new county records have also been added to the list as in Scriber *et al*. (1996) in Louisiana, Texas, Mississippi, Alabama, Arkansas, and Missouri (indicated in Fig. 2). As indicated in the Methods section, there are at least 432 additional counties (in the 9 states of Fig. 2), which document the *P. glaucus* species but fail to indicate if dark females occur.

The central latitudes and around the foothills of the Smokey Mountains, the frequencies of dark females has consistently hovered at more than 80%–98% (in most of Alabama, Georgia, Indiana, Kentucky, Louisiana, Mississippi, North and South Carolina, Ohio, Tennessee, and eastern Texas; Lambremont, 1954; Brower & Brower, 1962; Scriber *et al*., 1996). It had been assumed that in Alabama and Mississippi, essentially, only dark females existed (Mather, 1954), with only a single yellow female ever reported (Mather & Mather, 1958). However, in 1999, yellow females were collected in Lafayette County, in northern Mississippi by Cheryl Frankfater (4 yellow and 3 dark females in June; and 3 yellow and 4 dark females in August; pers. comm.; see Fig. 2).

### Preliminary evaluation of potential pleiotropic effects (physiological/ecological “costs”) of the dark gene (b+) trait

#### Size differences

A latitudinal trend of larger sizes at lower latitudes from Florida to smaller sizes in Alaska has persisted in the *P. glaucus* and *P. canadensis* populations for 40–50 years (Scriber, 1994; Lehnert *et al*., 2012), and, with the exception of local “climatic cold pockets” in Michigan with rapid warming during the summer (Scriber *et al*., 2014), the sizes at any given location have remained basically constant, even in the face of significant degree‐day thermal unit increases during the past 15 years. Subsamples of populations for which we have detected the recent declines in dark morph frequencies (Highlands Co, FL; Levy Co. FL; Clarke/Oglethorpe Cos. GA; Rabun and Habersham Co. GA & Macon Co NC; Lawrence & Gallia Cos. OH; Figs. 3–7) show that the sizes of dark morph females and yellow morph females are virtually identical in any particular year and location (Table 1). The seasonal increase in female size in summer versus spring generations is significant (4–8 mm in forewing lengths), but not between color morphs (Table 1).

#### Egg viability and fecundity

There were no significant differences between color morphs in egg viability for major populations in eight states (Table 2). Similarly, there were also basically no differences in female fecundity (total eggs produced) between color morphs. However, this fecundity measure of fitness is variable in our lab assays because of several unmeasured or uncertain factors, including the possibilities that: (1) field‐captured females may have differentially depleted their supply of fertile eggs since their last mating, before their capture and (2) potential differences among females may exist in willingness to oviposit in our plastic arenas (see Scriber, 1993).

Adult life span in the lab may not be a good reflection of field survival or age of first reproduction for females. However, preliminary field mark‐recapture data (Scriber *et al*., 1998a; Lederhouse & Scriber, unpublished) suggest that the life spans of dark and yellow morph females do not differ, but no data on age‐specific reproduction or fecundity in the field exist.

#### Postdiapause emergence delays

The date of postdiapause eclosion for *P. glaucus* is critically important to avoid potential extreme cold stress (if too early), and loss of mating/ reproductive potential (if too late). In thermally constrained regions (Scriber & Lederhouse, 1992; Scriber, 2002a), early emergence may be selected for, to permit completion of growth and pupation for that generation. The synchrony of males and females is also important. Controlled environment experimental results here indicate that over a very large range of temperatures, (11–29 °C) there were basically no differences in the time of eclosion (postdiapause) between daughters of yellow and dark females or between sons of dark or yellow mothers (Table 3). Marginally significant differences were only seen at 17 °C where adult daughters from dark females emerged a few days later (36.4 ± 0.9) than those from yellow mothers (32.4 ± 1.0 d), and at 29 °C (which is an exceptionally high temperature and unlikely to be encountered this early in the season) where the daughters of yellow mothers were a few days later to emerge than those dark mothers (Table 3). Postdiapause pupal developmental threshold differences between color morphs were basically nonexistent, with males and females (from yellow or dark morph mothers) all at roughly 10.2–10.5 °C (Fig. 9).

#### Host (oviposition) preferences

The host plant oviposition preferences were virtually identical for yellow and dark morph females from all locations tested (NC, GA, OH, PA; Table 4). The seven‐choice arenas included tulip tree (*Liriodendron tulipifera* of Magnoliaceae), hop tree (*Ptelea trifoliata* of Rutaceae), white ash (*Fraxinus americana* of Oleaceae), black cherry (*Prunus serotina* of Rosaceae), spicebush (*Lindera benzoin* of Lauraceae), *Rhamnus* spp. (Rhamnaceae), and quaking aspen (*Populus tremuloides* of Salicaceae). The rank order of preferences was also the same across all locations (see Mercader & Scriber, 2005, 2007).

#### Larval survival and growth rates

The ability of neonate larvae to survive on tulip tree (TT), black cherry (BC) and quaking aspen (QA) do not differ between offspring of different color morphs of *P. glaucus* (see Table 5; populations from VA, GA, and MO). The primary hybrid (F_1_) offspring of interspecific crosses using dark and yellow morph *P. glaucus* mothers from PA with *P. canadensis* fathers both exhibit excellent survival and growth, with those from dark mothers appearing to have a slightly higher survival on all three hosts, although with only four families, the differences did not reach statistical significance (Table 5). The total larval durations (neonate to pupae) for offspring of the two different color morphs were not significantly different at any of the four temperatures (28, 25, 22, 19 °C; all at 16 : 8 h photoperiod; Table 6).

#### Mid‐winter temperature stresses

Despite the fact that winter warming and chilling can differentially affect *P. glaucus* and *P. canadensis* (Kukal *et al*., 1991; Mercader & Scriber, 2008a; Scriber *et al*., 2012; Scriber, 2014), no differences in diapausing pupal survival between those from dark or yellow mothers was seen at any of the 4‐d mid‐winter thermal stresses imposed (–21, –18 °C, –13, or +20 °C; Table 7). The body water content and metabolic costs to diapausing pupae during diapause and spring emergence times were not different for dark and yellow morphs (Table 8). Extended periods of mid‐winter cold stress (10, 20, and 35 d at –18 °C) for diapausing pupae of yellow and dark female offspring produced no significant differences in survival or developmental time to adult eclosion (Table 9).

#### Male mate choice (sexual selection) discriminate against dark females

When offered field‐tethered pairs of size‐matched virgin females of both yellow and dark morphs, wild male mating preferences were basically frequency‐dependent (*P. glaucus* males preferred yellow females in 71% of copulations in southern Florida (Highlands Co.), where the yellow female frequency was 55%–70%; and *P. glaucus*. males preferred dark morphs for 76% of copulations in Ohio where population frequency was 75%–85% dark females; Table 10). In Levy County, Florida, the dark frequency was 45%–60% and the mating frequency preferences for dark were 50%–58% (Table 10).

In the Michigan hybrid zone and into the northern peninsula, where no dark females exist, *P. canadensis* males basically ignored dark *P. glaucus* females (H. Hereau and J.M. Scriber, unpubl. data). These hybrid zone males even preferred heterospecific yellow *P. glaucus* females over *P. canadensis* females of their own species (tethered, sized‐matched pairs; see Deering & Scriber, 2002). Males in the hybrid zone (where dark females are very rare) apparently avoid mating with dark females, with dark/yellow paired tether results in Michigan *P. canadensis* populations have shown only two males to copulate with the dark *P. glaucus* females (while 42 copulated with yellow *P. glaucus* females; Hereau and Scriber, unpubl. Data; Table 10).

#### Spermatophore counts as indirect measures of wild male preferences

The numbers of spermatophores in the bursa copulatrix of females as an index of mating frequencies among yellow and dark females of *P. glaucus* also (with the exception of the extremely worn and very oldest females) failed to show differences as the population ages (female wing wear serves as an index of age; Table 11, see also Aardema & Scriber, 2015). The extended analysis of spermatophore numbers of the 2002 Levy Co. Florida population illustrates similar age‐specific rates of mating for yellow and dark females (Table 12), with only the extremely oldest/worn dark females showing a slightly higher number of spermatophores than the similarly oldest yellow females.

**Table 11:** Characteristics (wing lengths, age, mating frequencies) of wild‐collected Levy Co. Florida *Papilio glaucus* females during 1 April to 14 April, 2002. Data presented as means, by date

	Dark morph females				Yellow morph females			
Date		FW		Spermat.		FW		Spermat.
	(*n*)	(mm)	Age	(*n*)	(*n*)	(mm)	Age	(*n*)
(2002)								
1 Apr	49	63.8	2.57	1.49	49	63.0	2.42	1.47
2 Apr	32	63.6	2.47	1.38	21	63.0	2.50	1.33
5 Apr	34	64.6	2.56	1.56	13	64.1	2.54	1.38
9 Apr	28	64.5	3.16	1.46	16	64.3	3.21	1.13
11–12 Apr	10	65.8	2.60	2.00	8	64.8	3.31	1.50
13–14 Apr	16	64.9	3.19	2.25	10	62.8	3.60	1.70

*t*‐tests show significance between dark morph and yellow morph females at 0.05 level for spermatophores only for 11–14th April.

Total captured (above) and sighted; 267 dark 454 total = 58.8% dark morph in 2002.

**Table 12:** Female mating status, Levy Co. Florida (during 1 April to 14 April 2002)

Age, or wing wear class of females	Color	*n*	Mean number spermatophores
1.0	Dark	1	1.00
	Yellow	1	1.00
1.5–2.0	Dark	41	1.34
	Yellow	32	1.22
2.5–3.0	Dark	98	1.51
	Yellow	54	1.54
3.5–4.0	Dark	29	2.10
	Yellow	30	1.40

Dark versus yellow mating frequencies were different (*P *= 0.05) for the very oldest/worn females (age class 3.5–4.0).

Spermatophores inside females sampled in Levy Co. Florida during these periods ranged from 1.38 to 2.60 for dark and 1.33 to 2.0 for yellow (Tables 11 and 12), suggesting a slight potential overall mating preference for dark females in this population (see Fig. 10). Further south at Highlands Co. Florida (Lederhouse & Scriber, unpublished) the mean number of spermatophores found in wild yellow females was 1.37 (*n = *119), which was not different than wild dark females with 1.40 (*n = *76).

### Recent frequency declines in dark morph females

While their forewing (body) size has remained constant during this recent 15‐year warming period across all latitudes for *P. glaucus* (Scriber *et al*., 2014), the frequency of dark females appears to have declined at key population sampling sites. At the southernmost populations in Highlands County, Florida (27.3°N latitude), it was seen that the frequency of dark females has dropped from the annual means of 40%–35% seen (during 1980–1999) to 19%–7% (2001–2009; Fig. 3). Similarly, at the Levy County site a bit further North (29.1°N latitude), the dark morph frequency dropped from roughly 80%–70% (1991–1995) to 60%–40% (2000–2011) and 24%–12% (2014–2017; Fig. 4). In Oglethorpe and Clarke Counties of northern Georgia (34.0 °N latitude, near Athens), the annual mean dark morph frequencies declined from 89%–88% (1984–2000) to 60%–45% (2003–2010) and to less than 30% in 2011 (Fig. 5). Smaller sample sizes nonetheless suggest that at the northern border of Georgia (Habersham and Rabun Counties) and adjacent Macon Co. North Carolina, the recent dark morph frequencies (50%–66%; 2002–2009) are lower than the 80%–90% averages observed previously during 1984–1992 (Fig. 6). They are considerably lower than the 97% dark frequency reported in 1959 in the adjacent Fannin and Union Counties (Brower & Brower, 1962). Although only a single year of sampling (2008) was completed in southern Ohio (Lawrence & Gallia Cos.) since the recent warming, the frequency of dark morph females was distinctly lower (40%) compared to the 85% average pre‐1998 (Fig. 7).

It was generally assumed that Mississippi and Alabama had only dark morph females (Mather, 1954). Only a single yellow *P. glaucus* female had ever been reported from Mississippi or Alabama (Mather & Mather, 1958). However, the new records of dark morphs in Lafayette Co. Mississippi, four yellow (and three dark) females were collected June/July and three yellow (and four dark) females of *P. glaucus* in August 1999 (Cheryl Frankfater, pers. comm.), suggest that a decline in dark frequencies (or increased yellow morph frequencies) may be geographically extensive (Fig. 2). The reasons for these declining trends in the frequencies of dark morph females are not clear.

### Dark morph (putative mimic) P. glaucus distribution closely corresponds to that of Battus philenor (model)

The known counties with verified records for dark morph females of *P. glaucus* are indicated from the Great Lakes region to the New England area in eastern United States (Fig. 1) These records are shown on the thermal landscape that reflects the average seasonal accumulations from 1980 to 1989 (which is only slightly warmer than 1970–1979 and 1960–1969 totals; see Scriber & Ording, 2005). Along the northern area, the boreal and temperate deciduous ecotone and the historical suture zone of hybrid interaction between *P. canadensis* and *P. glaucus* (Remington, 1968; Scriber *et al*., 2003) occupy the thermal landscape delineated by 2500–2700 degree‐days. It is also evident that the northern limits of dark morph females cooccur there as well (rarely has a single female seen anywhere with fewer than 2600 degree‐days; Figs. 1 and 2). This is supported by historical records for New York (Shapiro, 1974), Wisconsin (Ebner, 1970), Michigan (Neilsen, 1999), and the MA Statewide Butterfly Survey of 1986–1990 (as well as numerous personal communications to JMS). A single “blow‐in” was observed in Dickinson Co. in Michigan's Upper Peninsula (Scriber *et al*., 1998a,b) but is otherwise never been recorded in the northern area of the State of Wisconsin or Michigan's UP.

Extensive data collections across multiple transects of the hybrid zone illustrate the generally close correspondence of the northern distribution limits of the putative model (*Battus philenor*) and the dark (mimetic) *P. glaucus*. A similarly very close geographic correspondence of the *B. philenor* model and the mimetic form of *Limenitis* has been recently documented (Ries & Mullen, 2008). However, while dark females geographically seem to “track” the thermal isoclines in Minnesota, Iowa, and Western Wisconsin (Fig. 1), the pipevine swallowtail “model” does not occur there. *Battus philenor* is generally found as far north (or further) than dark *P. glaucus* females East of the Great Lakes region (Platt & Brower, 1968; Opler & Krizek, 1984; Hagen, 1990)

Unlike the polyphagous *P. glaucus* that feeds on plants from several families of angiosperms, *B. philenor* larvae only eat plants in the Aristolochiaceae family (Scriber, 1984). Such host specialization suggested that range limits for Aristolochiaceae host plants would closely reflect range limits for this pipevine swallowtail species. County records for the eastern U.S. species of Aristolochiaceae (*Aristolochia macrophylla* Lamarck), *A. tomentosa* Sims, *A. clematitis* L., and *A. serpentaria* L.) suggests that the northern limits of plants clearly correlate with that of the Aristolochiaceae‐specialized butterfly, *B. philenor*, from Missouri to New England. Western species of *Aristolochia* (e.g., *A. watsonii* Pfeifer; Crosswhite & Crosswhite, 1985) and Texas pine forest species (e.g., *A. reticulate* Nuttall; Scriber & Feeny, 1976; and *A. erecta* Englemann & Gray permit the existence of *Battus philenor* across southern Texas and the southwest and into California [Fordyce, 2000; Fordyce & Nice, 2003] beyond the range of *P. glaucus*) (Figs. 1 and 2).

#### Does predation actually select against yellow tiger striped females and favor survival of dark mimics?

While blue jays (Brower & Brower, 1962; Codella & Lederhouse, 1989, 1990) appear capable of discriminating between mimetic and nonmimetic patterns of *Papilio*, the extent of general bird predation (or lizard or invertebrate predation) has not been clearly demonstrated. Some preliminary experimental analyses of field predation were conducted using dark and yellow tethered females of *P. glaucus*. In an early‐afternoon assessments of *Anolis carolinensis* lizard predation in Highlands Co. Florida (Scriber *et al*., 1998a), both yellow and dark females were attacked when tethered near each other in pairs. In a follow‐up study of *Anolis* feeding on the “presumed model,” it was observed that the lizards repeatedly ate *Battus philenor* without any apparent hesitation or any ill‐effects (Mark Deering, pers. comm.).

Early morning bird predation assessments (*n = *5 pairs at Highlands Hammock, Florida; 6 pairs at Cedar Key, Levy Co. Florida) were made in 2006 (and *n = *57 pairs in 2008 at Levy Co.) using tethered pairs of dark and yellow females. As with the mate preference tethering (above), females were tied around the thorax between the forewings and hindwings with black thread so that they were able to fly on this tether and choose the resting site near where the thread was attached (using an alligator clamp).

In Florida, two types of tethering presentations were used in two years of evaluations. One was a paired tethering (one dark and one yellow morph tethered about 2 m apart along the edge of the woods). The other was with equal numbers of dark and yellow tethered individually along two sides of the road (butterfly flyway). In 2006, six adjacent pairs (1.5 m apart) of yellow and dark morphs were tethered for one night and morning at the Levy Co. site, and three dark and three yellow morphs were eaten. Although preliminary, these results do show that dark morphs are not immune from predation. Using “pairs” on opposite sides of the road (approximately 10 m apart) in Levy Co. Florida in the spring of 2008 additional replications of tethered color morphs were made (April 2, *n* = 17 pairs; April 3, *n* = 18 pairs; and on April 4, *n* = 22 pairs of dark and yellow morphs). The total numbers of tethered individuals in Florida missing (eaten) over this 3‐d period were eight yellow morphs and seven dark morphs (one dark, zero yellow on day 1; four dark, two yellow on day 2; and four dark, four yellow on the day 3). Again the predators did not appear to discriminate between the morph colors. Of 68 total pairs tethered out in Florida, missing or dead females were observed in 11 yellow and 10 dark females.

Butterflies tethered out in northern Michigan for two evenings (Aug. 9th and 10th; and checked in the mornings) suffered intense predation, were almost completely eaten, damaged severely or totally removed by the end of the 48 h. Of the 20 yellow morph *P. glaucus* females, 13 were totally missing, 6 were alive (some damage), and 1 was dead (partially eaten) on the tether. Of the 20 dark morph *P. glaucus* females, 15 were totally missing, 4 alive, and 1 dead on the tether.

#### Interspecific hybridization with western species (*P. eurymedon* and *P. rutulus*)

There are real costs to females in hybrid offspring due to Haldane Effects (Hagen & Scriber, 1995). While interspecific field mating preferences of western American *Papilio* were not examined in these studies (but see Brower, 1959), the relative physiological or genetic (Haldane) incompatibilities as reflected in sex‐ratios (see Hagen & Scriber, 1995) were similar for dark morph daughters and yellow morph daughters in each interspecific cross (nearly no daughters for *P. eurymedon* Lucas and *P*. *rutulus* Lucas pairings, and 25%–38% for *P. canadensis* pairings; Table 13). Genetic incompatibilities (Haldane effects; often greatest in the heterogametic sex as we have seen in female *Papilio*) in our interspecific primary (F‐1) hybrids were evident in the skewed sex‐ratios of crosses with a *P. canadensis* father (g × c), but not the reciprocal hybrid cross with *P. glaucus* males (c × g; Table 14). Most significant are the sex ratios for reciprocal hybrids between the *P. glaucus* and *P. canadensis* showing more sons (*n* = 66 families) than daughters (11 families) in families with *P. canadensis* fathers Table 14). Males are apparently buffered from such mortality (perhaps due to their homogamy; see Rockey *et al*., 1987a,b; also Presgraves, 2002). In contrast, sex ratios in 42 hybrid families are more balanced when the fathers were *P. glaucus* (Table 14).

**Table 13:** The relative numbers of sons and daughters in interspecific hybrids between *P. glaucus* females (both yellow and dark morphs) and *P. canadensis* (Pc), *P. rutulus* (Pr), or *P. eurymedon* (Pe) males. The heterogametic daughters from both dark (b+) and yellow (b–) mothers reflect strong Haldane effects, in all crosses regardless of mother color (b+, W‐linked)

Genotype	# Families	# Sons	# Daughters	Dead pupae†	%Female
Pg (Dark) × Pc	93	1162	699	679	37.5
Pg (Yellow) × Pc	22	152	51	131	25.1
Pg (Dark) × Pe	16	148	1	190	0.7
Pg (Yellow) × Pe	9	75	0	60	0.0
Pg (Dark) × Pr	13	198	7	161	3.4
Pg (Yellow) × Pr	13	164	3	286	1.8

^†^Most of these dead pupae were females (see Carter & Feeny, 1985; Scriber *et al*., 1990). *P. glaucus* dark females were reared from FL, GA, LA, OH, TX SC, IL, IN, WVA, southern MI, and southern WI. Yellow morphs were reared from WVA, southeast PA, and southern WI. *P. canadensis* were from northern WI, northern MI, and Alaska, and *P. eurymedon* & *P. rutulus* were from CA and WA (data from Scriber *et al*., 1990; see also Hagen & Scriber, 1995).

**Table 14:** Sex ratios of reciprocal primary hybrid families (mothers listed first, fathers last). Data of Hagen and Scriber (^*^1995) with 77 families and recent hybrid families (*n *= 49) for comparison

Reciprocal	(Fams)	>Sons	Equal	>Daughters	*P*
Hybrids					
Pg × Pc	39^*^	24	4	11	0.03
	45 recent	42	3	0	
	Total	66	7	11	
Pc × Pg	38^*^	14	10	14	0.83 ns
	4 recent	1	1	2	
	Total	15	11	16	

Note: ns = nonsignificant differences in number of sons and daughter.

Experimental backcross progeny show similar Haldane Effects (Table 15). The female pupae with recombined Z‐chromosomes that possessed either the *canadensis*‐like Ldh allozyme or the *canadensis*‐like Pgd allozyme (or both) entered a permanent diapause and died (Table 15). In recombinant backcrosses genes on the *P. canadensis* chromosome between Ldh and Pgd allozymes seem to disrupt development in combination with *P. glaucus* genes (Hagen & Scriber, 1995; Scriber, 2011).

**Table 15:** Intrinsic genetic selection against female hybrid recombinants with *P. canadensis‐*type allozymes. Data reflect the proportion of backcross female adults relative to males, with the same paternally inherited Pgd and Ldh alleles. Compare a recent backcross family 18006 segregation (shown at bottom; Pg mother × PgPc father; Ording & Scriber, unpubl. data) with seven backcross families (top; Hagen & Scriber, 1995). Numbers of offspring are indicated in parentheses

Alleles	Pgd‐100 (*glaucus*‐type)	Pgd‐125 (or Pgd‐80) (*canadensis*‐type)
Seven backcross families (Hagen & Scriber, 1995)		
Ldh‐100 (glaucus‐type)	1.09 (37/34)	0.35 (6/17)
Ldh‐80 (or Ldh‐40) (canadensis‐type)	0.61 (14/23)	0.03 (1/31)
Recombined hybrid backcross family #18006 (Ording & Scriber, unpubl)		
Ldh‐100	1.20 (18/15)	0.45 (5/11)
Ldh‐80 (or Ldh‐40)	0.67 (4/6)	0.14 (1/7)

## Discussion

### Pleiotropic physiological costs of melanism?

The potential physiological and ecological “costs” for melanism have been shown to be significant for some Lepidoptera (e.g., smaller size or lower tolerance to desiccation; Safranek & Riddiford, 1975), and for *Helicoverpa armigera* (Ma *et al*., 2008) melanism was associated with slower development in all life stages, smaller body size, lower fecundity and a lower reproductive rate. However, for *Papilio glaucus*, no obvious costs of female melanism (dark morph versus yellow morph females) were detected for a variety of life history factors.

The adult female size attained in natural populations has consistently shown no differences between dark and yellow morph females at any location from southern Florida to Michigan (Table 1; and Lehnert *et al*., 2012). Fitness costs associated with smaller size in insects may include reduced environmental stress buffering, poorer mating success, timing of adult emergences, and fecundity or egg viability (Ayres & Scriber, 1994; Chown & Gaston, 2010). However, no differences in total egg production (fecundity) nor viability of eggs was observed between dark and yellow morph females at locations in 8 different States (WI, MI, OH, IN, MO, VA, GA, and FL; Table 2). Oviposition preferences of dark and yellow morph females did not differ in seven‐choice oviposition arenas with key *Papilio* host plant species from Magnoliaceae, Rutaceae, Rosaceae, Oleaceae, Lauraceae, Rhamnaceae, and Salicaceae (Table 4; see also Mercader & Scriber, 2008b; Mercader *et al*., 2009). Neonate larval survival and growth rates on different hosts (Tables 5 and 6), the total developmental duration, and pupal postdiapause emergence times (Table 3) failed to uncover any costs of being melanic or having come from melanic mothers.

The experimental insertion of 4 d of mid‐winter (January) cold stress (*–*21 °C, –18 to 13 °C) and 4 d of warming stress (+20 °C) showed no differences in the pupal weight loss and survival of pupae from yellow mothers or dark mothers. This was observed both in families derived from the field in southeastern Pennsylvania (Lancaster Co.) and northern Georgia (Clarke Co.). However, the Georgia pupae from both color morphs exhibited high mortality at –21 °C (60%–80%) compared to those from Pennsylvania (0%–16%; Table 7). Despite differences between *P. canadensis* and *P. glaucus* pupal metabolism and stress resistance (Kukal *et al*., 1991; Mercader & Scriber, 2008a; Scriber *et al*., 2012), no differences were observed here in body water content or metabolic costs during winter diapause of *P. glaucus* pupae from dark or yellow mothers (Table 8). Extreme cold stress on diapausing pupae at mid‐winter (10, 20, and 35 d at –18 °C) also failed to illustrate any differences in the ability of dark or yellow morph offspring to develop and survive to adult eclosion in the Spring (Table 9). The adult emergence times from postdiapause pupae did not differ between offspring of dark morph and yellow morph mothers (Table 3), and the developmental thresholds were calculated to be virtually identical for males and females between these types (Fig. 9).

### Differential predation and melanism

While preliminary studies here suggest little differentiation in bird predation in Levy Co. Florida or Cheboygan Co. Michigan, it will require more extended studies during all local generations. More significantly, it would be especially important in the future to conduct additional field tethering studies of the two color morphs in areas with high populations of the *Battus philenor* model, as in the southern Appalachian Mts. and adjacent States (Brower & Brower, 1962; Scriber *et al*., 1996). Changes in the local population sizes (or density) of the model species is not well known from year to year or seasonally, but potential declines in the *B. philenor* populations of eastern North America could also be involved in the decline in dark morph frequencies over the past 15–20 years.

However, in March and April at Levy County in north in Florida, the decline in dark morph frequencies of *P. glaucus* seems unlikely due to any decrease in the model species, which has always been rare (since 1991, only one *B. philenor* was seen in 1995; two in 2003; two in 2013; three in 2017). Slightly skewed flight peaks for the model and mimics nevertheless suggest rarity for the model and fail to explain changes in dark morph frequencies of tiger swallowtail females in Levy County, FL (Fig. 4).

If avian predation is significantly avoided by mimetic dark morph females, then it is feasible that the increased numbers “intermediate,” “cinnamon” or “dusted” phenotypes observed at higher pupal development temperatures (Ritland, 1986; Scriber *et al*., 2009a,b) could partially explain why recent declines in dark morph frequencies have occurred. The nonmimetic dusty “intermediate” phenotypes would not have mimetic advantage and might be eaten more readily than if they were typical dark morphs. These intermediate phenotypes nonetheless have the dark morph genotypes (with both the Z‐linked enabler gene, s–, and the W‐linked melanism gene, b+) and thus, avian selection pressure on them would reduce the genetic potential for dark morphs in the population (Fig. 10). However, why recent climate warming seems to be directly correlated with declining dark morph frequencies at all populations studied from Florida north to Michigan remains unclear (Figs. 3, 4, 5, 6, 7).

### Recent frequency declines across the range of P. glaucus

Although a few stray dark morph *P. glaucus* females have been recently reported north of the historical hybrid zone (Scriber *et al*., 1998b; Blomberg & Herig, 2014; Fig. 1), the higher latitude/altitude distribution of these putative mimics has remained basically similar for the past four to six decades (Scriber *et al*., 1996; Scriber, 2011). Interspecific genetic introgression has been documented for various species diagnostic traits (especially autosomal, but also certain Z‐linked traits; including, oviposition preferences, Pgd‐100 allozyme alleles; and the melanin “enabler,” s–, Hagen & Scriber, 1991; Mercader *et al*., 2009) recently moving northward and upward across the hybrid zone. However, others such as the Z‐linked allozyme Ldh‐100, W‐linked melanism (b+), and mitochondrial DNA have not (Donovan & Scriber, 2003; Stump *et al*., 2003; Scriber *et al*., 2008; Kunte *et al*., 2011; Scriber, 2011; Zhang *et al*., 2013; Ryan *et al*., 1917).

During the past 10–15 years, a general decline in dark morph female frequencies in eastern North America has been reported here, from Florida to Ohio (Figs. 3–7). The continuing high frequency of dark morph females in the Smoky Mts. (and Alabama, Mississippi) has been presumably related to the high densities of the model species, *Battus philenor* (Brower & Brower, 1962). However, this has not been experimentally evaluated to date. Many untested ecological/evolutionary assumptions about the center versus the edges of species ranges need serious evaluation (Sagarin *et al*., 2006). Studies across the entire species ranges are needed since differential selection pressures exist at high versus lower latitudes of the range (Hampe & Petit, 2005; Scriber *et al*., 2008) as well as locally (e.g., in climatic cold pockets; Scriber *et al*., 2014).

**Figure 8:**
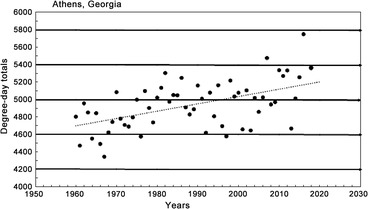
The Annual degree‐day totals (base 50 °F) at Athens Georgia (1960–2016). It was reported that at least 57 consecutive days exceeded 90 °F in July and August of 2016.

Dark morph female frequencies were very high for *P. glaucus* populations in the southern Appalachian Mountains (97% dark in northern Georgia at the border with TN and NC; Brower & Brower, 1962) and we have seen this frequency generally range from 80% to 90% in northern Georgia during the years 1984–1992). Further to the North dark morph female frequencies since the late 1990s (and before) have declined in northern Georgia (Figs. 5 and 6) and southern Ohio (Fig. 7). Potential reasons for such declines in the recent decade are not entirely clear. However, the significant increases in annual summer growing season degree‐day accumulations (1960–2016) may play some unknown role (Fig. 8). For example, in 2017 the Athens region of northern Georgia experienced the warmest summer in the previous 57 years (Fig. 8) and the dark morph frequency has dropped from 97% to less than 60% in the last decade (Fig. 5).

**Figure 9:**
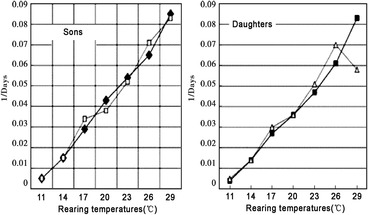
Postdiapause developmental rates and thresholds for adult emergences of sons and daughters of dark morph mothers (solid black symbols) and for offspring of yellow mothers (open symbols).

**Figure 10:**
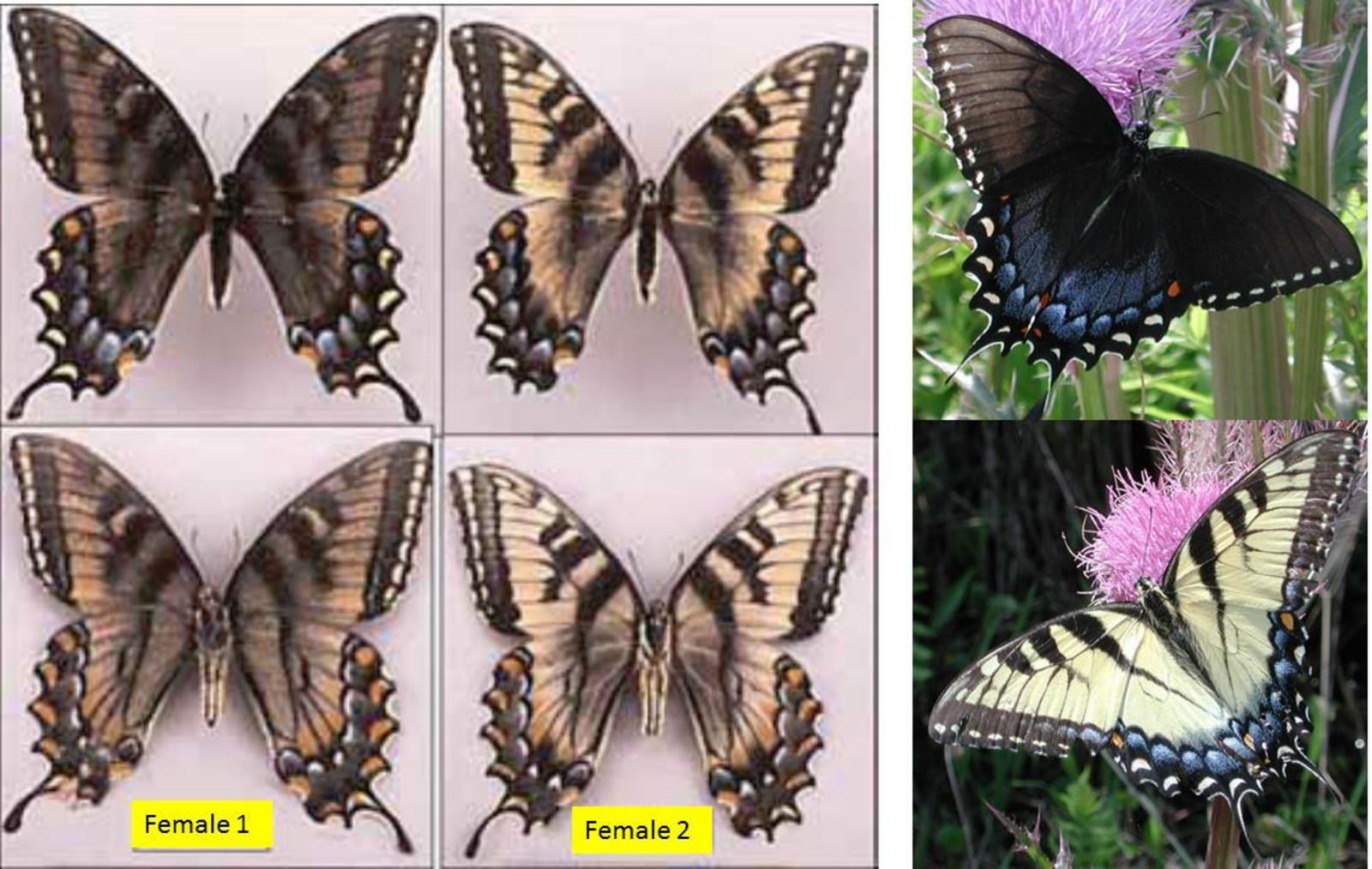
Two “intermediate” (dusted or cinnamon‐looking) phenotypes of dark morph females (Scriber *et al*., 1996; Scriber *et al*., 2009b; Carpenter, 2014). Compare normal dark and yellow morphs at right.

### Differential mate preferences for yellow/dark morphs (sexual selection)

Our preliminary tethered female field studies to date (Table 10) suggest that the mating preference for yellow or dark females has been basically density dependent. In Michigan, dark females drifting northward in the hybrid zone appear not be recognized by *P. canadensis* or hybrid males despite the fact that such males prefer yellow *P. glaucus* females over their own size‐matched *P. canadensis* females (Deering & Scriber, 2002). Areas with high frequencies of yellow females such as southern Florida (Highlands Co.) and northern Michigan (Isabella Co.) reflect preferences for the yellow female of the two‐choice field mating preference assays. Areas with high frequencies of dark females reflect preferences for dark females (including southern Ohio and northern Florida (Levy Co.; Table 10).

However, the experimental preferences of free‐flying males was clearly for yellow females when tethered with dark in a two‐choice array at this Highlands Co. FL location (Table 10), suggesting random, frequency‐dependent preferences rather than a balancing selection pressure for yellow as opposed to dark mimetic types as suggested by Burns (1966). While dark morph frequencies have recently declined, it is not evident that changing mate preferences would drive this trend. However, increasingly higher frequencies of yellow morphs at any location might in some way be enhanced by faster population growth of yellow morphs with subsequent preferences for yellow females also increased, possibly as a result.

Mating “attractiveness” of female (*glaucus* and *canadensis) hybrids* are hypothesized to be largely X‐linked. Sexually selected traits are often sex‐linked (Prowell, 1998; Reinbold, 1998; Shaw & Parsons, 2002), but this possibility has not been evaluated for reciprocal Pc × Pg hybrid daughters for free‐flying hybrid zone (or *P. appalachiensis*) males. However, such genetic factors may play key roles in hybrid introgressiuon, and hybrid speciation (see below).

### Differential hybrid introgression of Z‐linked enabler and W‐linked melanism gene (Pleiotrophic issues)

Recombinant hybrid populations with mixtures of Z‐chromosome diagnostic traits and “late” (July) flights in univoltine obligate diapausers (Hagen & Lederhouse, 1985; Rockey *et al*., 1987a,b) occur in central New York State and Vermont Battenkill River populations (Fig. 1) as well as in the hybrid species, *P. appalachiensis* (Scriber & Ording, 2005; Ording *et al*., 2010; Kunte *et al*., 2011). Mixtures of recombining Z‐linked traits may occur throughout the historical hybrid zone (Scriber, 1994, 2011; Putnam *et al*., 2007; Cong *et al*., 2015).

Sex ratio analyses have also been very informative in regard to the Haldane and X‐effects in *Papilio* species showing decreased survival with greater phylogenetic differentiation (such as *P. glaucus* hybridization the Mexican *P. alexiares*, and western species *P. eurymedon, P. rutulus*, and *P. multicaudatus* Kirby; Hagen & Scriber, 1989, 1995; Scriber *et al*., 1990a,b). However, genetic incompatibilities in such interspecific hybrids and backcrosses does not appear to differ for dark versus yellow morph *P. glaucus* mothers (Table 13; Hagen & Scriber, 1995). Thus the costs of genetic compatibilities are not directly related to the W‐linked melanism gene.

Endogenous selection against hybrids may be due to deleterious X–Y chromosome, X‐autosome, Y‐autosome, or mt‐DNA/ heterospecific chromosome interactions (see Jiggins *et al*., 2001a,b; Coyne & Orr, 2004; Gemmell *et al*., 2004). Perhaps these genetic incompatibilities may be involved in differential endogenous selection against female color morphs (or their offspring) in the hybrid zone (Scriber *et al*., 2003, 2008).

Our studies show that strong selection has been observed against the recombinant backcross daughters, which possess one or both of the *P. canadensis*‐type Z‐linked allozyme alleles (Ldh and Pgd). When such offspring are expressed as a proportion of females, relative to males, with the same two paternally inherited allozyme alleles (paternal haplotypes), the four recombinant backcross X‐linked allozyme genotypes clearly show a shortage of females in the eight backcross families (Table 14).

These data suggest that either a region of *P. canadensis* X ( = Z)‐chromosome between Ldh and Pgd (or separate regions near each locus) contains the genes that disrupts pupal development in combination with *P. glaucus* genes (“Haldane Effect”). Diapause (Z‐linked, od+) and mimicry (W‐linked, b+) genes may serve as potential causes of hybrid inviability in Tiger Swallowtails: If W‐linked b+ dark allele in *P. glaucus* coevolved closely with its Z‐linked enabler (s–) genes, then it may not function properly with homologous genes from other species (including *P. canadensis*). The Z‐linked suppressor gene (s+) in *P. canadensis* may suppress dark expression, but may also cause other developmental dysfunction involving the *P. glaucus* Y‐chromosome (the only females affected in viability were those with *P. glaucus* W‐chromosomes).

While the Haldane Effect on diapausing female pupae (permanent diapause = death) is not simply a result of the negative pleiotropic effects of possessing the b+ gene for melanism, different causes may exist regarding “Haldane effects” for sterility versus inviability (Presgraves 2002). Resolving these possibilities require more molecular markers. In addition, potential direct pleiotropic effects of the W‐linked b+ gene have not been fully examined.

### P. appalachiensis and P. glaucus from Pendleton Co. West Virginia

It has been shown the hybrid species (Mountain Swallowtail, *P. appalachiensis*) contains mostly *P. canadensis*‐derived genome (about 72%; Cong *et al*., 2015). However, the *P. glaucus*‐derived Z‐linked dark morph enabler (s–), which is close to the 6PGD allozyme locus, has shown up in the hybrid species (Pavulaan & Wright, 2004; Scriber & Ording, 2005; Scriber, 2011; Cong *et al*., 2015), despite the fact that neither of these Z‐linked traits were detected or reported in Kunte *et al*. (2011).

The origins of *P. appalachiensis* as a mountain version of the delayed false‐second generation hybrid swarm late flights (univoltine “LF” in mid‐July) seen in the cooler side of the hybrid zone has been suggested (Scriber & Ording, 2005; Scriber *et al*., 2008). These postdiapause emergence delays may have provided virtually immediate temporal reproductive isolation from both parental species (Ording *et al*., 2010; Scriber, 2014). A similar postdiapause delay in adult emergences (as in hybrid *Papilio* “late” flights; Ording *et al*., 2010; Cong *et al*., 2015) resulting in temporal reproductive isolation, occurs in hybrid ecotypes of the European Corn Borer moth, and also are regulated by Z‐linked “postdiapause delay” factors (Dopman *et al*., 2010; Wadsworth *et al*., 2013; Levy *et al*., 2014; Scriber, 2014; Wadsworth & Dopman, 2015).

### Other potential causes of melanism costs or decline in dark frequencies in Papilio

While no obvious differences (e.g., “costs” of melanism) were detected in many important life history traits described above, there are many other potential pleiotriophic costs (or perhaps benefits) of melanism that have not yet been studied. For example, one very intriguing concept is that the melanic forms of Lepidoptera may have higher parasitoid resistance (Verhoog *et al*., 1996) or higher disease resistance to viruses (Gershenson, 1994) or fungi (Wilson *et al*., 2001). To our knowledge, this has not been evaluated in any species of *Papilio* or other swallowtail butterflies of the Papilionidae. Melanism in some *Spodoptera* species is associated with a genetic trade‐off between two immune system components (upregulating phenoloxidases and downregulating lysozymes) with negative impact on developmental rates (Cotter *et al*., 2008). In addition, stressful conditions such as low nutritional quality of plants can cause phenotypic‐specific mortality in adult melanics (Zvereva *et al*., 2002; Ethier *et al*., 2015).

Also, an unexamined possibility exists that the recent climate warming across the species range of *P. glaucus* may somehow present subtle physiological challenges to dark females compared to yellow females where elevated temperature extremes, higher daily means, or greater daily variances occur (see also Tesar & Scriber, 2003; Scriber & Sonke, 2011). Perhaps, for example, the dark melanic morphs may have a greater difficulty thermoregulating in hotter conditions.

It is also feasible that ecological selection by predators of nonmimetic yellow females may include “intermediate” brownish‐yellow (cinnamon) individuals (see Scriber *et al*., 1987, 1996; Carpenter, 2014, Fig. 10), which are known to result from pupal development and adult emergence at higher temperatures (Ritland, 1986). Such climate‐enhanced predation on these “cinnamon”‐colored females (Scriber *et al*., 2009b) would eliminate genes for melanism (b+ on the W‐chromosome) from these populations (Scriber *et al*., 1996) and therefore could result in reduced frequencies of melanic females. Such natural selection could partially explain the decline in dark morph frequencies during the recent climate warming of the recent two decades (Figs. 3, 4, 5, 6, 7). Other Lepidoptera also display cold‐induced increases in melanism (Sourakov, 2015). This possibility, and other potential differential mortality or subtle impacts on the eggs, larvae, pupae and adults of dark morph offspring (relative to those of yellow morphs) deserves additional study in the field as well as controlled environment lab conditions.

## Summary/conclusions

The North American geographic range limits and frequencies of the dark (mimetic) morph females of *Papilio glaucus* had been basically constant for the last few decades of the 20th century. However, starting in 1997, a continuing or accelerating decline in dark morph frequencies (and increase in yellow tiger‐striped morphs) has been documented at several long‐term study populations in Highlands County of Florida, Levy County of Florida, Clarke & Oglethorpe Counties in Georgia, adjacent counties in northern Georgia (Rabun & Habersham) and Macon County North Carolina, and in southern Ohio (Gallia and Lawrence Counties). Other southern areas that had been essentially all dark morphs have recently experienced an increase in yellow morphs. For example, in 1999 several yellow females of *P. glaucus* were reported in both the June and the August flights in northern Mississippi (the first yellow females reported in the State since the single record in 1958; Mather, 1954; Mather & Mather, 1958).

Several potential factors potentially contributing to these dark morph frequency declines across eastern North America were investigated here, including ecological, physiological, behavioral, and genetic selection pressures. However, the potential pleiotrophic costs to *P. glaucus* females of being all dark (possessing both the W‐linked melanism gene (b+) and the Z‐linked enabler gene (s–)] instead of being the tiger‐striped yellow morph were not evident in any of the traits examined here). These include: 1) adult size, 2) fecundity, 3) egg viability, 4) larval survival, 5) larval growth rates, 6) total developmental durations, 7) short‐term mid‐winter thermal stresses (warm and cold) on diapausing pupae, 8) postdiapause pupal development rates and thresholds, and 9) oviposition preferences in multichoice arrays. Other, unknown pleiotrophic effects that might reduce fitness of the dark morph may be involved and should be investigated (e.g., differential egg, larval and adult stress from extreme, or continuously high, field temperatures; differential disease and parasite tolerances, etc.).

While male mating preferences for yellow morph females was previously suggested as a possible factor resulting in a balanced polymorphism with the advantage of dark mimetic females of *P. glaucus*, it has been shown that males do not always favor yellow females in their courtship and matings. Evidence from spermatophore counts in wild females and direct mating behavior using tethered dark and yellow female pairs was reviewed and expanded. The results clearly suggest that the mating behavior by *P. glaucus* males with the two color morphs is random (or frequency dependent, locally), making it highly unlikely that it would be responsible for the female frequency changes.

The W‐linked dark gene (b+) in females appears to move more slowly than most traits on the Z‐chromosome which are shown to extensively recombine and introgress via interspecific hybridization (Putnam *et al*., 2007; Scriber *et al*., 2008; Scriber, 2011; Cong *et al*., 2015; Ryan *et al*., 2017, 2018b). What actually stops this W‐linked melanism gene (b+) from moving further up in latitude or altitude (in contrast to the Z‐linked melanism enabler, s– gene) has not been determined. Lack of selection favoring dark mimics where the putative “model” becomes scarce seems unlikely to account for the steep clinal limits for dark morphs, but it still may play some role (but see Ries & Mullen, 2008; Pfennig & Mullen, 2010). For example, the rare model concept in Highlands County Florida was supported by the low (6%–8%) frequencies of the mimetic *P. glaucus* dark morph in the 1950s but not supported by the higher frequencies (30%–50%) from 1960 to 1987 (Lederhouse & Scriber, 1987b).

Genetic incompatibilities in offspring of hybrid matings can be a strong selection pressure contributing to Haldane Effects, where the heterogametic female hybrid offspring suffer greater mortality than the homogametic hybrid males (Haldane, 1922). However, the W‐linked melanism factor (b+) is not likely to be responsible for this effect since reduced survival of hybrid females was observed in daughters of both dark and yellow morph mothers. The constraints on mitochondrial DNA movement northward basically stop at the same latitudes/altitudes as does the W‐linked melanism gene (Stump *et al*., 2003; Kunte *et al*., 2011; Scriber, 2011), but these are not necessarily linked in function or occurrence (Andolfatto *et al*., 2003).

The ecological (mimetic) advantages of dark morph females in regions with high frequencies of the *Battus philenor* (model) species has been postulated as a major selection pressure, and dark morph frequencies have been the highest in such areas where the model is most abundant (Brower, 1958; Brower & Brower, 1962). The geographic distribution of the dark morph *P. glaucus* mimic and the *B. philenor* model are in general agreement, but the mimic exceeds the range of the model extensively in the Midwest, west of Indiana. Preliminary bird predation experiments (with small sample sizes) using tethered dark and yellow morph females in the field suggest that differential predation rates on yellow and dark females may not exist in Florida or Michigan (near the dark range limits). However, studies have not been done in the latitudes in between where the model abundance is highest. Nonavian predators such as spiders, mantids and Anole lizards also appear to be nondiscriminatory between dark and yellow morph *P. glaucus*, although increased replication is needed before any rigorous conclusions about potential mimic (or even model) avoidance by these predators can be evaluated properly.

After two centuries, the geographic extent and frequencies of dark and yellow morph females of *P. glaucus* in eastern North America remains an interesting ecological and evolutionary phenomenon. The recent discovery of this color dimorphism also occurring in the recombinant homoploid hybrid species (*P. appalachiensis*) in the area where the parental species (*P. canadensis* and *P. glaucus*) overlap provides unique opportunities for study of genetic introgression, recombinant hybrid divergence, homoploid hybrid speciation, and evolutionary genomics (Scriber *et al*., 2008; Kunte *et al*., 2011; Cong *et al*., 2015; Ryan *et al*., 2017; 2018b).

## Disclosure

The author declares no bias nor conflicts of interest in this research.

[Corrections added on 28 February 2019, after first online publication:

1. The following citations have been added to the article text:
  • Dubovskiy *et al*., 2013 on page 2, column 1, line 6
  • Scriber, 2002b, 2010; Scriber & Gage, 1995 on page 7, column 2, line 24
  • Hagen & Lederhouse, 1985 on page 21, column 2, line 6
  • Fordyce, 2000 on page 18, column 2, line 7
  • Figure 10's citation has been added on page 20, column 2, line 3 and on page 22, column 2, line 26
2. The following references have been deleted:
  • Remington, C.L. (1956)
  • Swenson, N.G. and Howard, D.J. (2004)
3. The following changes should be made to the text:
  • On page 11, column 1, in the heading ‘Updated distribution records for dark morph females (since regional climate warming 1997–2014)’, 1997–2014 has been amended to 1997–2018.
  • In Table 8, “*n*” should be added in column 2.
  • In the references list, Dubovshiy *et al*., 2013 has been corrected to Dubovskiy *et al*., 2013, and DOI has been amended to ‘280, 10.1098/rspb.2013.0584’.
  • On page 18, column 2, line 7, the citation ‘Fordyce *et al*., 2003’ has been amended to ‘Fordyce & Nice, 2003’.
